# Supplementary material for: Neuronal RNAi and oxygen-sensing circuit shape germline resilience to heat stress
Source: Curr Biol. 2026 Jul 20;36(14):3566–3579.e5. doi: 10.1016/j.cub.2026.06.016 (PMC13399994; doi:10.1016/j.cub.2026.06.016)
Supplement: Document S1. Figures S1–S4 [file mmc1.pdf]

Current Biology, Volume *36*

Supplemental Information

Neuronal RNAi and oxygen-sensing circuit shape germline resilience to heat stress

Chee Kiang Ewe, Hanna Achache, Hanna Schön, Leonid Kontorovich, Guy Teichman, Shir Weiss, Anna Mogilevskaya, Myriam Valenski, Sarit Anava, Rutwik Bardapurkar, Hila Gingold, Rachel Posner, Olga Antonova, Mario de Bono, Yonatan B. Tzur, and Oded Rechavi

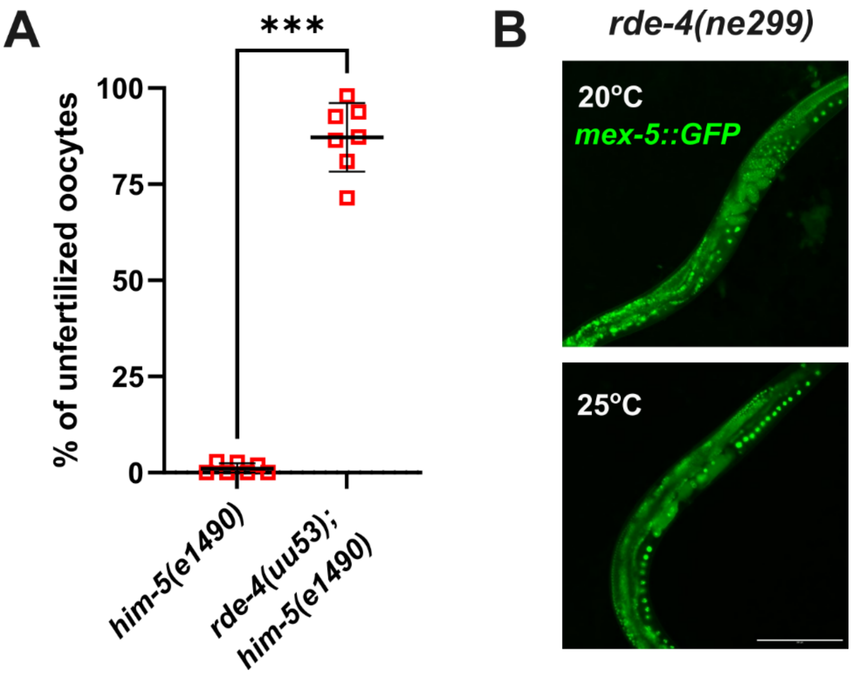

Figure S1. RDE-4 promotes reproduction at high temperature. Related to Figure 1. (**A**) *rde-4(uu53)* mutants exhibit severe loss of fertility at 25 ^{o}C. Error represents mean ± SD. Statistical significance was determined by Mann-Whitney test. *** $p < 0.001$. (**B**) *rde-4(ne299)* day 2 adult shows accumulation of unfertilized oocyte stacked in the gonad at 25 ^{o}C, but not at 20 ^{o}C. The germline is marked by *gfp* driven by *mex-5* (RNA-Pol II) promotor.

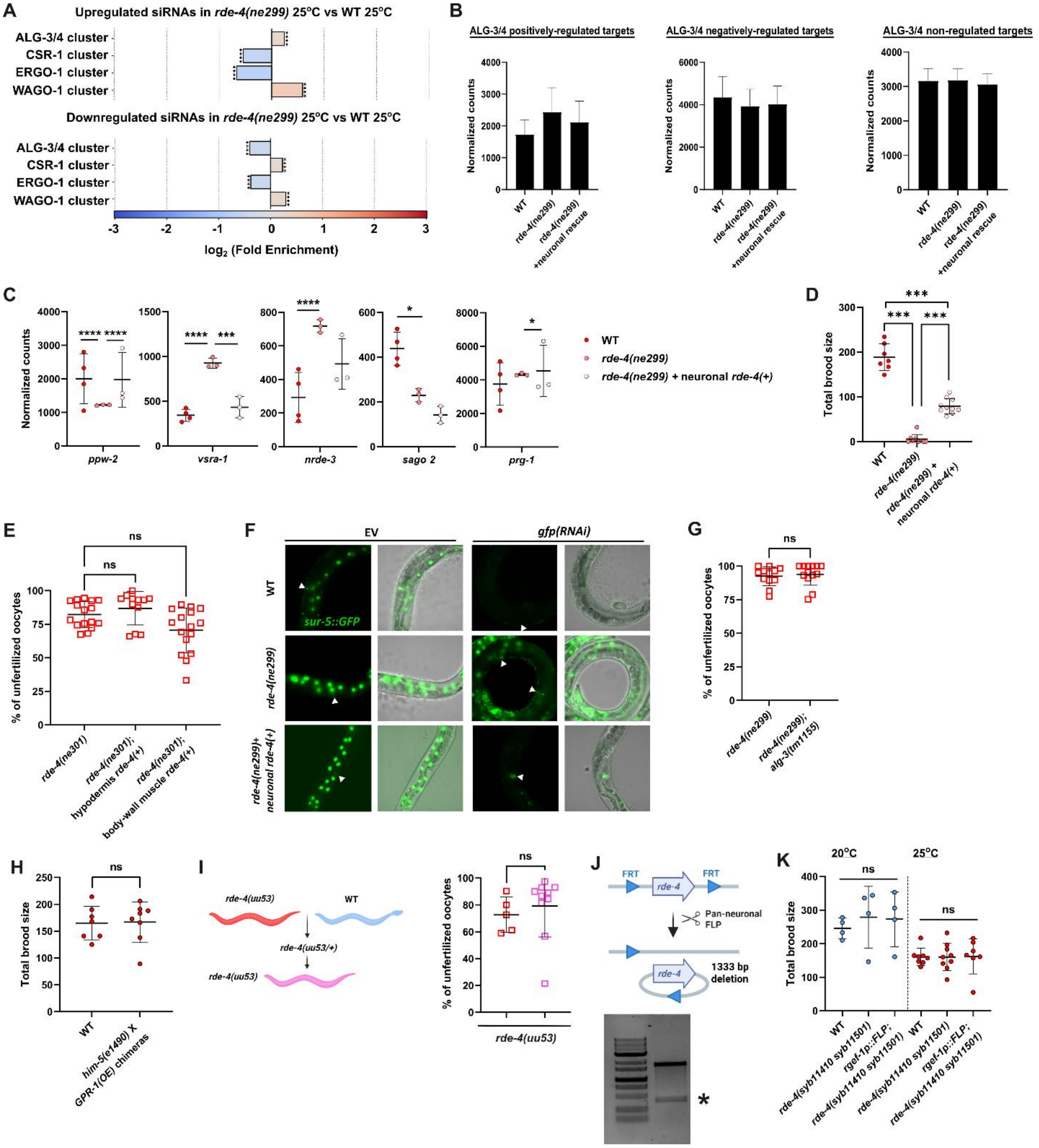

Figure S2. Neuronal RDE-4 promotes sperm development. Related to Figure 2. (**A**) Different classes of endo-siRNAs are differentially expressed in *rde-4(ne299)*. ALG-3/4-class siRNAs tend to be upregulated, whereas CSR-1–class siRNAs tend to be downregulated in *rde-4(ne299)* compared to wild type. (**B**) Expression of different classes of ALG-3/4 targets, as defined by Conine et al, in wild type, *rde-4(ne299)*, and *pigSi3(Psng-1::rde-4); rde-4(ne299)* mutants. Error represents mean ± SEM. (**C**) Misexpression of AGO genes in *rde-4(ne299)* is rescued by neuronal *rde-4(+)* in some cases. Relative log expression (RLE) is shown. Statistical significance was determined by DESeq2. (**D**) *rde-4(ne299)* mutants show a reduced brood size compare with

wild type, and this is rescued by *pigSi3(Psng-1::rde-4)*.(**E**) Expressing *rde-4* in hypodermis (driven by *nas-9* promotor) or body-wall muscle (drive by *myo-3* promotor) does not rescue *rde-4(ne301)* fertility defects. Note that *ne301* and *ne299* contain identical lesion. (**F**) Neuronal *rde-4(+)* does not rescue RNAi-defective phenotype of *rde-4(ne299)*. Arrows indicate spermatheca. (**G**) Loss of *alg-3* does not affect *rde-4(ne299)* fertility. These strains contain *him-5(e1490)* mutation. (**H**) F1 chimeras generated by crossing GPR-1-overexpressing hermaphrodites with *him-5(e1490)* males does not show altered brood size. (**I**) Homozygous *rde-4(uu53)* segregated from heterozygous mothers show severe fertility defects. (**J**) Schematic diagram depicting FRT/FLP-mediated excision of neuronal *rde-4*. Asterisk denotes the deleted *rde-4* allele identified by PCR. (**K**) Deleting *rde-4* in neurons using the FRT/FLP system does not affect brood size. For (D), (E), (G), (H), (I), and (K), error represents mean ± SD and statistical significance was determined by Mann-Whitney tests. Multiple comparison corrections were applied where appropriate. ns $p > 0.05$; * $p \leq 0.05$; *** $p < 0.001$; **** $p < 0.0001$.

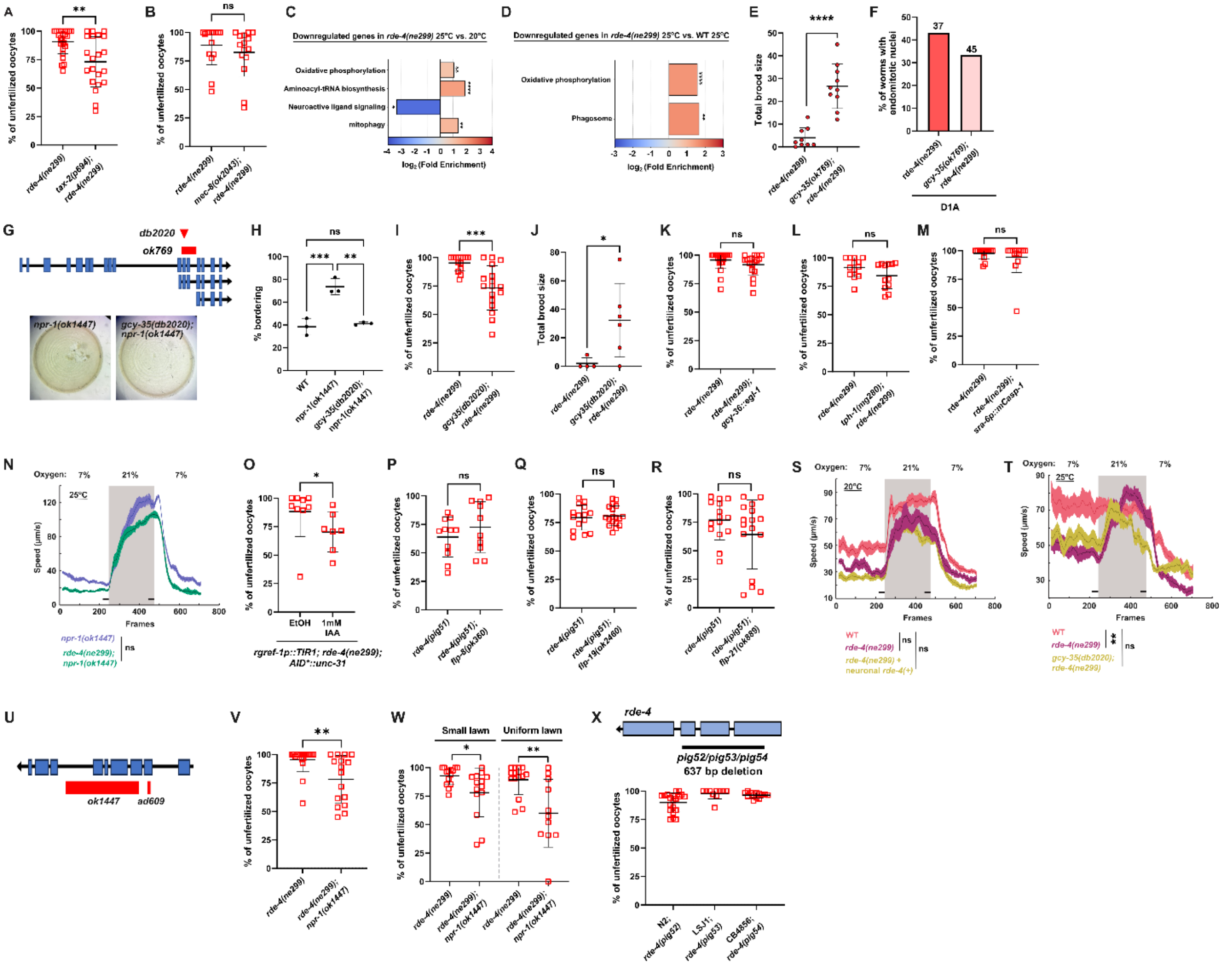

Figure S3. Neuronal sensory and NPR-1 affects fertility. Related to Figure 3. (**A**) Loss of *tax-2* partially rescues fertility defects in *rde-4(ne299)* at 25 ^{o}C. (**B**) Knocking out *mec-8* does not affect *rde-4(ne299)* fertility. (**C and D**) KEGG pathway analysis reveals that mitochondrial genes tend to be downregulated in *rde-4(ne299)* grown at 25 ^{o}C compared to either *rde-4(ne299)* at 20 ^{o}C or wild type at 25 ^{o}C. Differential gene expression analysis includes RAPToR age estimates as covariate. (**E and F**) Deleting *gcy-35* increases brood size and reduces the accumulation of endomitotic nuclei. D1A = day-1 adults. (**G and H**) Inserting a universal knock-in cassette (*db2020*) causes loss of function of *gcy-35*, which inhibits aerotaxis behavior of *npr-1(ok1447)*. (**I and J**) *gcy-35(db2020)* rescues fertility defects in *rde-4(ne299)*. (**K**) Ablating *gcy-36*-expressing neurons does not affect *rde-4(ne299)* fertility. (**L**) Eliminating *tph-1* does not rescue *rde-4(ne299)* sterility. (**M**) Genetically ablating ASH neurons does not impact *rde-4(ne299)* fertility. (**N**) *npr-1(ok1447)* mutants show increased responses to 7 % and 21 % oxygen at 25 ^{o}C. This is not affected by the loss of *rde-4*. (**O**) Depleting UNC-31 by AID partially restore *rde-4(ne299)* fertility. (**P-R**) Knocking out *flp-8*, *flp-19*, or *flp-21* does not affect *rde-4(pig51)* fertility. Error represents mean ± SD. (**S**) *rde-4(ne299)* and *pigSi3(Psng-1::rde-4); rde-4(ne299)* mutants show similar oxygen response as wild type at 20 ^{o}C. (**T**) Loss of *gcy-35* rescues oxygen response of *rde-4(ne299)*. (**U**) Schematic diagram depicting the positions and molecular nature of the *ok1447* and *ad609* mutations in *npr-1*. (**V**) Deleting *npr-1* partially rescues *rde-4(ne299)* fertility. (**W**) Deletion of *npr-1* rescues *rde-4(ne299)* fertility on both small

‘simple’ lawns and uniform lawns. (**X**) Deleting *rde-4* in CB4856 and LSJ1 causes severe loss of fertility at 25 ^{o}C, similar to that observed in N2. Error represents mean ± SD. For (N), (S), and (T), n = 7-9 assays, 20-25 animals per assay. Solid lines indicate average speed and error indicates SEM. Black horizontal bars indicate time intervals used for statistical tests. For (A), (B), (E), (H-T), (V) and (W), statistical significance was determined by Mann-Whitney tests. Multiple comparison corrections were applied where appropriate. ns $p > 0.05$; * $p \leq 0.05$; ** $p < 0.01$; p *** < 0.001; **** $p < 0.0001$.

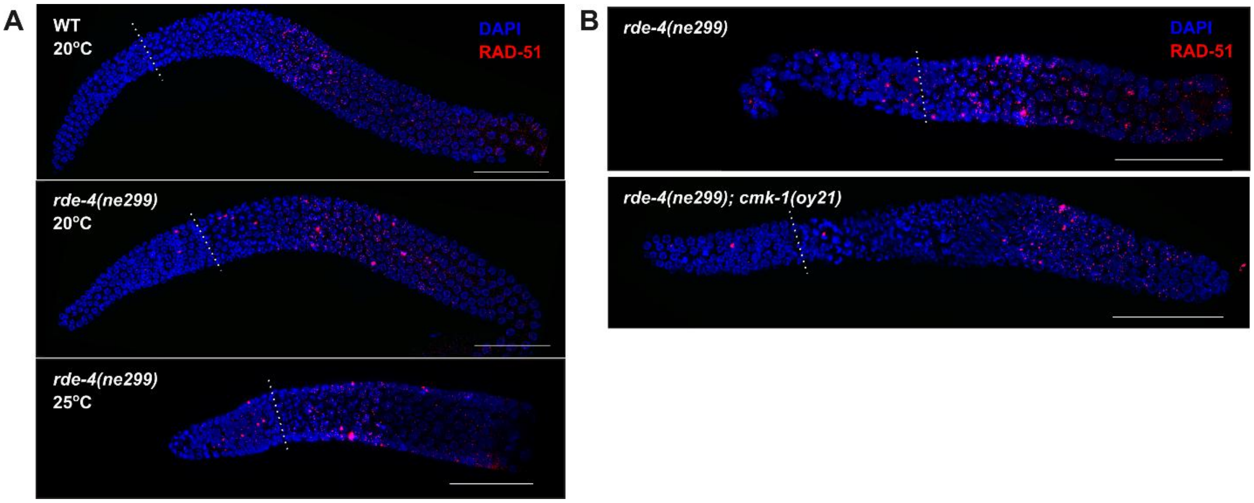

Figure S4. Neuronal sensory affects germline integrity. Related to Figure 4. (**A**) *rde-4(ne299)* grown at 25 °C, but not 20 °C, shows increased DSBs in the gonad. (**B**) Knocking out *cmk-1* reduces DSBs in *rde-4(ne299)* at 25 °C. White dotted lines indicate mitosis to meiosis transition. Antibody staining was performed on day-1 adults. Scale bar = 50 µm.
