# Supplementary material for: Neuronal RNAi and oxygen-sensing circuit shape germline resilience to heat stress
Source: Curr Biol. 2026 Jul 20;36(14):3566–3579.e5. doi: 10.1016/j.cub.2026.06.016 (PMC13399994; doi:10.1016/j.cub.2026.06.016)
Supplement: Document S2. Article plus supplemental information [file mmc5.pdf]

Current Biology

Article

Neuronal RNAi and oxygen-sensing circuit shape germline resilience to heat stress

Graphical abstract

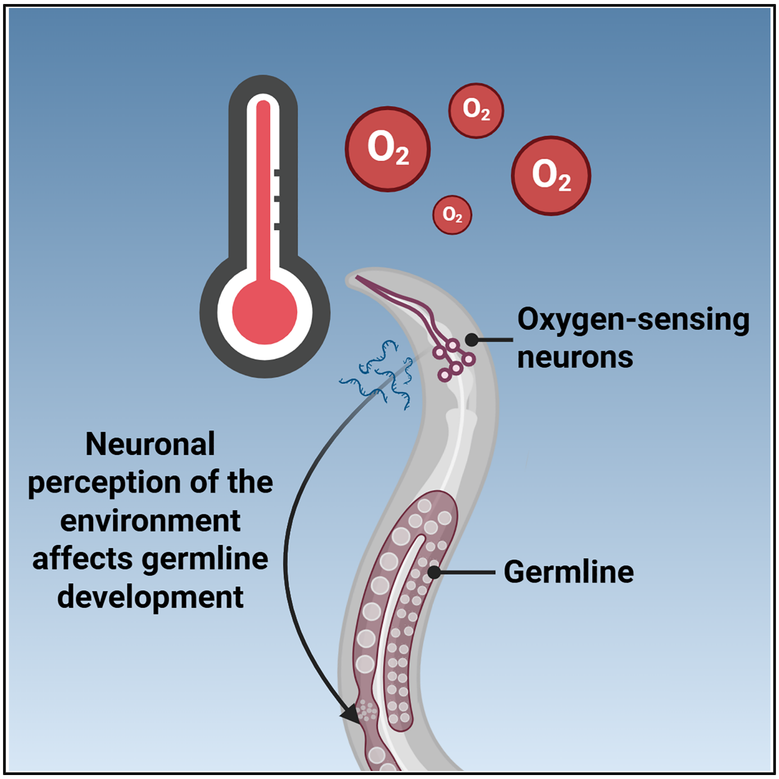

Highlights

- dsRNA-binding protein RDE-4 is required for germline thermotolerance
- Neuronal RDE-4 promotes germline thermotolerance
- Blocking oxygen sensing promotes germline development in *rde-4*-deficient animals

Authors

Chee Kiang Ewe, Hanna Achache, Hanna Schön, ..., Mario de Bono, Yonatan B. Tzur, Oded Rechavi

Correspondence

ethanewe@gmail.com (C.K.E.), tzur@mail.huji.ac.il (Y.B.T.), odedrechavi@gmail.com (O.R.)

In brief

Ewe et al. demonstrate that neuronal small RNAs can modulate germline development under heat stress in *Caenorhabditis elegans*. They further show that disrupting the oxygen-sensing neural circuit promotes germline thermotolerance, linking environmental perception to developmental outcomes and highlighting a role for RNAi in integrating environmental information to regulate reproductive robustness.

Ewe et al., 2026, Current Biology *36*, 3566–3579

July 20, 2026 © 2026 The Author(s). Published by Elsevier Inc.

https://doi.org/10.1016/j.cub.2026.06.016

CellPress

CellPress
OPEN ACCESS

Current Biology

Article

Neuronal RNAi and oxygen-sensing circuit shape germline resilience to heat stress

Chee Kiang Ewe,[1,4,*] Hanna Achache,[2,4] Hanna Schön,[3] Leonid Kontorovich,[1] Guy Teichman,[1] Shir Weiss,[1] Anna Mogilevskaya,[2] Myriam Valenski,[2] Sarit Anava,[1] Rutwik Bardapurkar,[1] Hila Gingold,[1] Rachel Posner,[1] Olga Antonova,[1] Mario de Bono,[3] Yonatan B. Tzur,[2,*] and Oded Rechavi[1,5,*]

[1]School of Neurobiology, Biochemistry and Biophysics, Wise Faculty of Life Sciences and Sagol School of Neuroscience, Tel Aviv University; Chaim Levanon Street 55, Tel Aviv Yafo 6997801, Israel

[2]The Alexander Silberman Institute of Life Sciences, The Hebrew University of Jerusalem, Givat Ram, Jerusalem 9190401, Israel

[3]Institute of Science and Technology Austria (ISTA), Am Campus 1, Klosterneuburg 3400, Austria

[4]These authors contributed equally

[5]Lead contact

*Correspondence: ethanewe@gmail.com (C.K.E.), tzur@mail.huji.ac.il (Y.B.T.), odedrechavi@gmail.com (O.R.)

https://doi.org/10.1016/j.cub.2026.06.016

SUMMARY

Thermal pollution, whether local or driven by global warming, threatens biodiversity in part through its detrimental effects on reproduction. Non-coding small RNAs (sRNAs) are crucial for maintaining germline developmental robustness under heat stress. Remarkably, we uncovered that neuronal sRNAs regulate germ cells' thermotolerance, affecting both spermatogenic and oogenic germlines in a cell-non-autonomous manner. Furthermore, we demonstrate that, in RNAi mutants, an oxygen-sensing neural circuit, modulated by neuropeptide signaling, antagonizes germline maintenance, likely reflecting the nematode's innate association of reduced oxygen levels with food availability and reproductive permissive environments. Finally, we provide evidence that laboratory-domesticated alleles of oxygen-response genes encoding neuropeptide receptor NPR-1 and hexacoordinated globin GLB-5 compromise germline thermotolerance. Hence, our findings raise the possibility that sensory perception, independent of direct environmental change, modulates germline integrity, highlighting a novel mechanism by which neural circuits integrate environmental information to safeguard reproductive fitness in fluctuating environments.

INTRODUCTION

Reproductive health is strongly influenced by environmental factors. Mass extinctions are happening on an unprecedented scale, driven, in large part, by rising global temperatures that cause widespread fertility loss. Climate change is linked to declining male and female fertility from plants to mammals, posing a significant challenge for future generations.[1,2] How do organisms endure and adapt in such difficult times?

Non-coding small RNAs (sRNAs), including siRNAs, miRNAs, and piRNAs, together with Argonautes (AGOs), play conserved roles in germline development and fertility.[3] In flies and mammals, loss of PIWI proteins leads to germ cell loss and transposon derepression.[4–6] In mice, endo-siRNAs are critical for oogenesis, while miRNAs and piRNAs are essential for multiple aspects of spermatogenesis.[7–11] Similarly, in the nematode *C. elegans*, sRNAs play key roles in germ cell development.[12–17] Given the potential of sRNA/AGO pathways to drive plastic gene regulatory programs, it is of great interest to understand how they regulate fecundity in changing environments.

Growing evidence pointed to the roles of somatic tissues—especially reproductive support cells—in transmitting environmental signals to the germline, in part by regulating RNA payload in germ cells.[18–21] For example, in flies, exosomes derived from the secondary cells in the accessory gland fuse with mature sperm and facilitate reproductive success.[19] In mammals, the epididymal epithelium may load RNAs onto maturing sperm via exosomes and microvesicles. Evidence suggests that paternal diet may affect RNA production in the epididymis and, in turn, reprogram sperm epigenome, enabling intergenerational inheritance of environmental signals.[20–23]

RESULTS

Endo-siRNAs protect sperm thermotolerance

TRBP2 is a dsRNA-binding protein essential for Dicer-mediated miRNA production in vertebrates.[24] In flies, its homolog R2D2 and Loqs-PD, together with Dicer, facilitate siRNA loading onto RNA-induced silencing complex (RISC).[25,26] In *C. elegans*, the TRBP2 homolog RDE-4 is critical for exogenous RNAi and antiviral defense by binding long dsRNAs and interacting with DCR-1/Dicer, DRH-1/RIC-I, and the AGO RDE-1.[27–30] RDE-4 is also required for the biogenesis of endogenous siRNAs and ensures their proper loading onto AGOs.[31,32] Central to this study, previous work uncovered an important role of RDE-4 in embryogenesis during heat stress, with the loss-of-function mutants exhibiting temperature-sensitive developmental defects and laying arrested embryos at 25°C.[33]

Here, we similarly found that *rde-4(–)* mutants are fertile at 15°C and 20°C; however, at 25°C, they lay unfertilized

This is an open access article under the CC BY license (http://creativecommons.org/licenses/by/4.0/).

CellPress
OPEN ACCESS

oocytes—large, dark, rounded cells with prominent nuclei—that are readily distinguishable from unhatched embryos (unfertilized oocytes: 80.6%; n = 500) (Figures 1A–1C). Unless stated otherwise, we scored eggs and unfertilized oocytes laid by day-2 adults at 25°C (see STAR Methods). Arrested unfertilized oocytes usually build up in the proximal gonad (stacked appearance) and may undergo meiotic maturation in the absence of sperm and become endomitotic, resulting in polyploidy and nuclear hypertrophy.[34] We observed this phenotype across three different alleles of *rde-4*: *ne299* (A123→STOP), *uu53* (925 bp deletion), and *pig51* (637 bp deletion), but not in wild-type animals (unfertilized oocytes: 0.06%; n = 236) (Figures 1A–1D and S1). We found that the fertility defects in *rde-4(−)* under heat stress stem from sperm failure, as crossing *rde-4(−)* to wild-type males rescued the phenotype (Figure 1E).

Given the sperm defects observed in *rde-4(−)* hermaphrodites (Figures 1A–1E), we wondered whether *rde-4(−)* also plays a role in male sperm development. To enrich for males, we introduced a *him-5* mutation, which induces a high frequency of X chromosome nondisjunction without compromising sperm morphology and functions.[35] In *him-5(−)* males, major sperm protein (MSP), which is essential for sperm functions in nematodes, is packed into fibrous bodies and localized to membranous organelles; on the other hand, its organization is severely disrupted, and MSP remains diffused in *rde-4(−);him-5(−)* males at 25°C (Figure 1F). In addition, we observed pronounced chromosomal abnormalities in spermatocytes from *rde-4(−);him-5(−)* males (Figure 1G). Because *fog-2(−)* mutants lack self-sperm and rely on mating for reproduction, *fog-2(−)* females crossed with *him-5* males restored fertility. However, *rde-4(−);him-5(−)* males failed to sire progeny at 25°C (Figure 1H). Together, these results indicate that RDE-4, functioning in the endo-siRNA pathway, is required for the thermotolerance of sperm in both hermaphrodites and males.

Neuron-to-germline communication affects sperm development in animals lacking *rde-4*

C. elegans contains a highly elaborate sRNA/AGO machinery, consisting of at least 19 AGOs that act sequentially to mediate potent gene silencing responses in diverse biological contexts.[36–38] In the endogenous siRNA pathway, the enhanced RNAi (ERI) complex produces 26G sRNAs using mRNAs as templates. The 26G sRNAs are then loaded onto primary AGOs–ERGO-1 in oocytes and embryos, and ALG-3/4 in sperm–which recruit RNA-dependent RNA polymerases (RdRPs) RRF-1 and EGO-1, leading to the production of abundant secondary 22G sRNAs.[12,39,40] In sperm, WAGO-10 and CSR-1a bind 22G sRNA and function downstream of ALG-3/4 to regulate spermatogenesis.[12,13]

RDE-4 is required for the full production of 26G endo-siRNAs, although it is dispensable in certain cases.[32,41,42] In this study, we performed sRNA-seq using a 5′-phosphate-independent protocol (see STAR Methods) and found that siRNAs (antisense reads without applying filters on 5′ nucleotide composition) targeting spermatogenic genes are upregulated in *rde-4(−)* mutants grown at 25°C compared with 20°C (Figure 2A). Notably, at 25°C, upregulated siRNAs in *rde-4(−)* relative to wild type are enriched for those targeting sperm genes, male-enriched genes, and certain known ALG-3/4-class sRNAs (Figures 2B and S2A).

We recently demonstrated that neuronal RDE-4 may alter germline gene expression and trigger transgenerational behavioral changes.[43] Intriguingly, here, we found that expressing *rde-4* in neurons as a single-copy MosSCI transgene under the control of a pan-neuronal promoter (*pigSi3[Psng-1::rde-4::SL2:yfp]*) could largely restore the expression of sperm siRNAs, many of which are associated with ALG-3/4, in *rde-4(−)* mutants (Figures 2B–2D). ALG-3/4 may mediate either negative or positive gene regulation of their targets[44]; consistent with this, we observed aberrant upregulation of many spermatogenic transcripts in *rde-4(−)* (Figures 2E and S2B), while genes that are known to be negatively regulated by ALG-3/4 are downregulated in *rde-4(−)* animals (Figure S2B). In addition, we detected misregulation of several other germline AGOs, including *csr-1* and *wago-10*, and RdRP genes (Figures 2F and S2C). These expression patterns were at least partially rescued by neuronal *rde-4(+)* (Figures 2E, 2F, S2B, and S2C). Together, our findings suggest that neuronal RDE-4 may regulate germline sRNA pathways cell-non-autonomously.

Strikingly, expression of neuronal *rde-4(+)* partially rescued the heat-induced fertility defects observed in *rde-4(−)* mutants (unfertilized oocytes: 89.5% in *rde-4(ne299)* versus 50.6% in *rde-4(ne299)* + neuronal *rde-4(+)*) (Figures 2G–2I and S2D). In contrast, hypodermal or muscle-specific rescue of *rde-4* driven by *nas-9* or *myo-3* promoter did not show an effect (Figure S2E). By performing smFISH and RNA-seq on isolated gonads, we previously confirmed that the neuronal *rde-4(+)* transgene is not mis-expressed in the germline.[43] Importantly, similar to *rde-4(−)* mutants and in contrast to wild-type animals, *rde-4(−)* mutants carrying the neuronal *rde-4(+)* transgene are not responsive to exogenous dsRNA targeting *gfp* expressed in sperm (Figure S2F). Hence, these findings indicate that the observed effects are not artifacts caused by transgene misexpression in the germline but reflect a *bona fide* cell-non-autonomous role of RDE-4 in regulating sperm heat tolerance. This neuron-to-sperm communication appears to be independent of SID-1, a conserved dsRNA-selective importer required for systemic RNAi (Figure 2J).[45]

ALG-3/4 are required for sperm development at elevated temperature: single *alg-3* or *alg-4* mutants show a severely reduced brood size at 25°C, whereas double mutants are completely sterile.[12] Our mRNA and sRNA sequencing experiments provided evidence consistent with elevated ALG-3/4 activity in *rde-4(−)* mutants (see above), and this is restored by neuronal *rde-4(+)*, raising the possibility that sperm development is sensitive to ALG-3/4 dosage such that either too little or too much is detrimental. To test this, we removed *alg-3* in the *rde-4(ne299)* mutants; however, this did not rescue fertility (Figure S2G). These results suggest that RDE-4 controls sperm development through additional AGOs that we did not test, or that neuronal *rde-4(+)* regulates fertility via an sRNA-independent mechanism, and the changes in sRNA profiles reflect the consequence, rather than the cause of restored fertility.

To provide additional support for our conclusion regarding RDE-4-dependent neuron-to-sperm communication, we performed mosaic analysis by inducing uniparental isodisomy. To achieve this, we crossed *rde-4(ne299);him-5(e1490)* males to

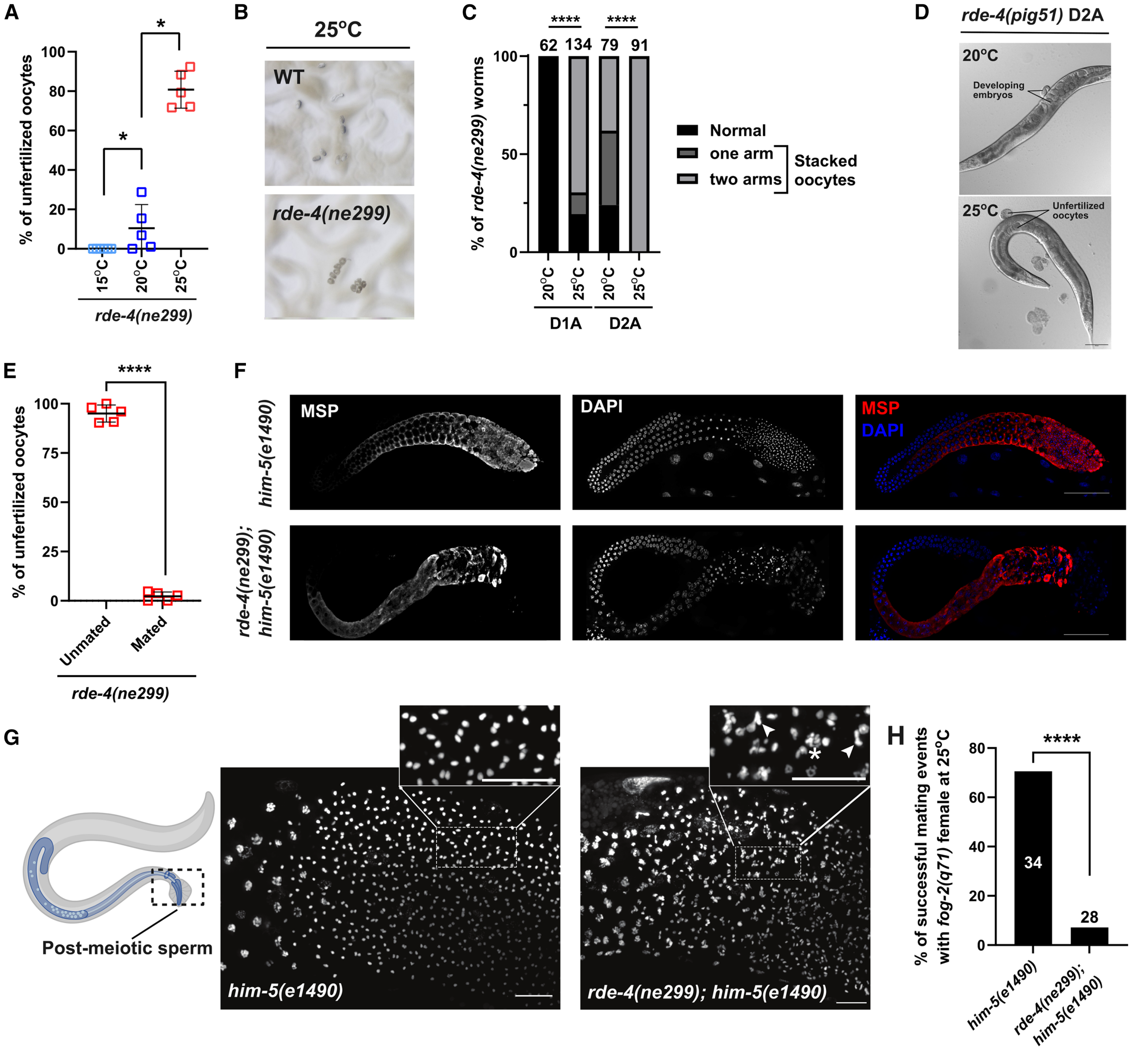

Figure 1. Loss of RDE-4 compromises sperm thermotolerance

(A–D) *rde-4(ne299)* shows temperature-sensitive fertility defects.

(A) L4 larvae were transferred from 20°C to 15°C or 25°C. The fertility of day-2 adults was scored. *$q \leq 0.05$ by Kruskal-Wallis test followed by pairwise Mann-Whitney tests.

(B) At 25°C, *rde-4(ne299)* animals lay unfertilized oocytes, which are morphologically distinct from fertilized embryos.

(C) Unfertilized oocytes tend to accumulate and stack within the gonads of *rde-4(ne299)* at 25°C. The presence of stacked unfertilized oocytes in one ("one arm") or both ("two arms") gonadal arms was quantified. D1A: day-1 adults; D2A: day-2 adults.

(D) *rde-4(pig51)* shows similar phenotypes to *rde-4(ne299)*. Scale bars, 100 μm. D2A: day-2 adults.

(E) The fertility defects of *rde-4(ne299)* at 25°C are rescued by crossing with wild-type males. In these experiments, L4 hermaphrodites were incubated with males at 25°C for ~24 h. Mated hermaphrodites were then transferred to fresh plates, and the numbers of eggs and unfertilized oocytes laid were scored. ****$p < 0.0001$ by Mann-Whitney test.

(F) MSP expression and localization are severely disrupted in the male gonad in *rde-4(ne299);him-5(e1490)* at 25°C. Scale bars, 50 μm.

(G) Sperm from *rde-4(ne299);him-5(e1490)* exhibit chromosomal abnormalities. Arrows mark chromosomal bridges; the asterisk indicates decondensation of DNA. DAPI and antibody staining were performed on day-1 adults. Scale bars, 10 μm.

(H) *rde-4(ne299);him-5(e1490)* males fail to mate with *fog-2(q71)* female at 25°C. Mating efficiency was estimated by scoring the presence of fertilized eggs in *fog-2(p71)* hermaphrodites after ~24 h of incubation with males.

For all relevant panels, error represents mean ± SD.

In (C) and (H), numbers indicate the total number of animals scored. ****$p < 0.0001$ by Fisher's exact tests.

See also Figure S1.

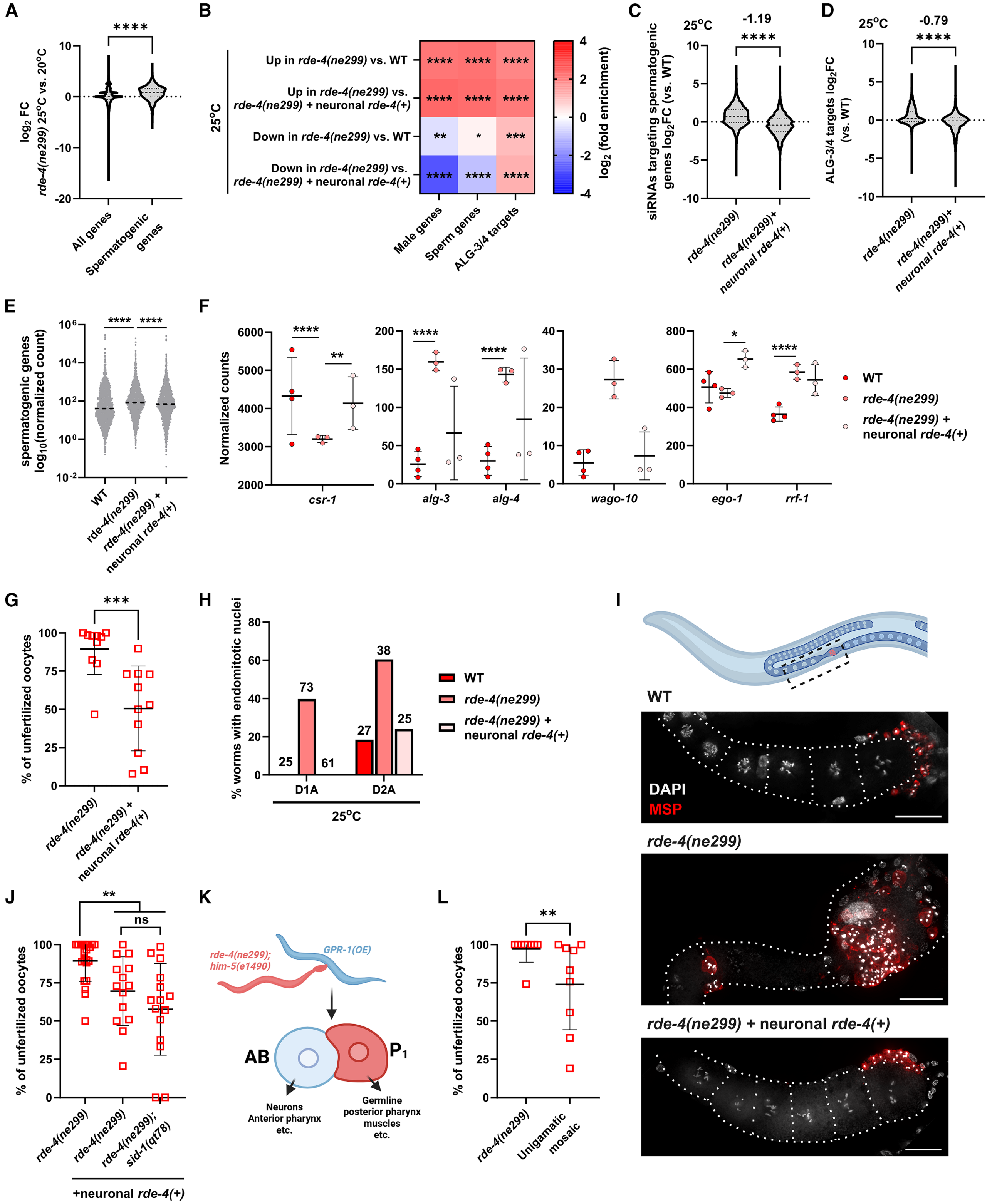

Figure 2. Neuronal RDE-4 promotes sperm thermotolerance

(A) siRNA targeting spermatogenic genes are upregulated in *rde-4(ne299)* grown at 25°C compared with 20°C. All sequencing experiments were performed on day-1 adults.

(legend continued on next page)

hermaphrodites overexpressing GPR-1, a conserved microtubule force regulator. This manipulation causes premature segregation of maternal and paternal chromosomes in the pre-cleavage embryos, resulting in non-Mendelian partitioning of genetic material between the first two blastomeres: AB and P_1. In this system, the anterior AB contains exclusively maternally derived chromosomes, while the posterior P_1 carries only paternally derived chromosomes.[46,47] As nearly all neurons are derived from AB,[48] they inherit the wild-type *rde-4* from the hermaphrodite, whereas the germline, which arises from the P_1 lineage, carries the *rde-4(ne299)* mutation from the male (Figure 2K). We found that these mosaic animals exhibit milder fertility defects compared with *rde-4(−)* mutants at 25°C (unfertilized oocytes: 97.1% in *rde-4(ne299)* versus 74.0% in *rde-4* chimeras) (Figure 2L). In our control experiments, chimeras generated by crossing *him-5(e1490)* males with GPR-1-overexpressing hermaphrodites produced brood sizes similar to those of wild type (Figure S2H). These results support our earlier findings that neuronal sRNAs promote sperm thermotolerance (Figures 2G–2I). Importantly, this effect cannot be attributed to maternal provision, as *rde-4* homozygous mutants derived from heterozygous mothers still display severe fertility defects (Figure S2I). Finally, we inserted FRT sequences at the 5′ and 3′ ends of the *rde-4* locus using CRISPR-Cas9 and excised the flanked sequence by neuronal expression of FLP recombinase (*rgef-1p::FLP*) (Figure S2J).[49] Loss of neuronal *rde-4* in an otherwise wild-type background did not overtly affect brood size at either 20°C or 25°C (Figure S2K). These results indicate that germline development is predominantly regulated by germline sRNAs, with neuronal RNAi playing a cryptic modulatory role that becomes apparent in response to genetic perturbation, such as loss of *rde-4* in the germline, and environmental challenge, such as heat stress.

Oxygen-sensing neural circuit negatively impacts germline development

Previous studies showed that RDE-4 and endo-siRNAs are required for various neuronal processes.[50–53] Importantly, we recently demonstrated that *rde-4(−)* mutants exhibit defective chemotaxis toward various volatile and soluble attractants at 25°C, but not at 20°C.[43] This defect is partially rescued by neuronal expression of *rde-4(+)* (Data S1).[43] Hence, neuronal RDE-4 is important for the detection of various environmental cues, especially in the presence of heat stress.

Given the roles of RDE-4 in sensory signaling, we tested whether blocking sensory perception affects sperm development. Interestingly, eliminating *cmk-1*, a gene encoding the homolog of CaMKI, which modulates sensory gene expression, partially rescues *rde-4(−)* sterility at 25°C (unfertilized oocytes: 96.3% in *rde-4(ne299)* versus 58.3% in *rde-4(ne299);cmk-1(oy21)*) (Figure 3A). We observed similar effects when we knocked out *tax-2*, which encodes a subunit of cyclic nucleotide (cGMP)-gated channel that is required for many sensory responses (unfertilized oocytes: 83.7% in *rde-4(ne299)* versus 38.0% in *tax-2(ok3403);rde-4(ne299)*) (Figures 3B and 3C). A partial loss-of-function allele, *tax-2(p694)*,[54] produced a similar, albeit weaker, effect (unfertilized oocytes: 90.8% in *rde-4(ne299)* versus 73.2% in *tax-2(p694);rde-4(ne299)*) (Figure S3A). These results indicate that the sensory inputs normally inhibit sRNA-mediated sperm heat tolerance.

Next, we asked which sensory modalities might regulate sperm development. Given that sterility in *rde-4(−)* mutants is temperature-sensitive, we first eliminated three guanylyl cyclases (GCs)—GCY-8, CGY-18, and GCY-23—which function specifically in AFD neurons, the primary thermosensory neurons in *C. elegans*.[55] While we previously showed that removing *gcy-8/18/23* downregulates AGO genes and disrupts heritable gene silencing in the germline,[56] we found that blocking heat sensation had no effect on the fertility of *rde-4(−)* mutants (Figure 3D). Similarly, impairing gustatory signaling by removing CHE-1, a C2H2-type zinc finger TF required for specification of the ASE gustatory neurons,[57,58] or disrupting general chemosensation by knocking out the G protein α subunits GPA-3 and ODR-3,[59] also did not impact fertility (Figure 3D). In addition, eliminating MEC-8, which plays a role in amphid cilia fasciculation and mechanosensory neuron development,[60,61] had no observable effect (Figure S3B).

C. elegans has strong behavioral responses to the gases oxygen and carbon dioxide, which are highly variable in its natural habitats such as soil, compost heaps, and rotting substrates. Environmental oxygen is primarily detected by the AQR, URX, and BAG neurons in the head, and the PQR neuron in the tail, with additional contributions from other neurons.[62–64] Mitochondrial activity modulates cellular oxygen consumption, affecting behavioral and physiological responses to environmental oxygen levels.[65,66] We performed differential expression analyses that include RAPToR age estimates as a covariate to control for confounding differences

(B–D) siRNAs targeting male-enriched genes, spermatogenic genes, and some ALG-3/4-class sRNAs (i.e., sRNAs mapped to ALG-3/4 target genes)[37] tend to be upregulated (DESeq2 q < 0.05) in *rde-4(ne299)* versus wild type at 25°C, which is rescued by *pigSi3[Psng-1::rde-4]*. The numbers in (C) and (D) indicate the differences in average $\log_2$FC.

(E) Spermatogenic transcripts are upregulated in *rde-4(ne299)* (average $\log_2$FC = 0.75) and restored by neuronal *rde-4(+)* (average $\log_2$FC = −0.25).

(F) Expression of AGO and RdRP genes is dysregulated in *rde-4(ne299)*, which is rescued by neuronal *rde-4(+)* in some cases. Relative log expression (RLE) is shown. *q ≤ 0.05; **q < 0.01; ****q < 0.0001 by DESeq2.

(G–I) Neuronal *rde-4(+)* partially rescues fertility defects in *rde-4(ne299)* at 25°C. The numbers in (H) indicate the total number of animals scored. D1A: day-1 adults; D2A: day-2 adults. (I) *rde-4(ne299)* day-1 adult carries endomitotic nuclei and shows disrupted MSP expression at 25°C. These phenotypes are rescued by neuronal *rde-4(+)*. Scale bars, 20 μm.

(J) The effects of neuronal *rde-4(+)* are not affected by the loss of *sid-1*.

(K) Schematic of mosaic uniparental inheritance induced by GPR-1 overexpression.

(L) Wild-type *rde-4* in the neurons of mosaic animals partially rescues fertility defects.

For all relevant panels, error represents mean ± SD. Not significant, $p > 0.05$; *$p \leq 0.05$; **$p < 0.01$; ****$p < 0.0001$ by Mann-Whitney tests. Multiple comparison corrections were applied where appropriate.

See also Figure S2 and Data S1, S2, and S3.

CellPress
OPEN ACCESS

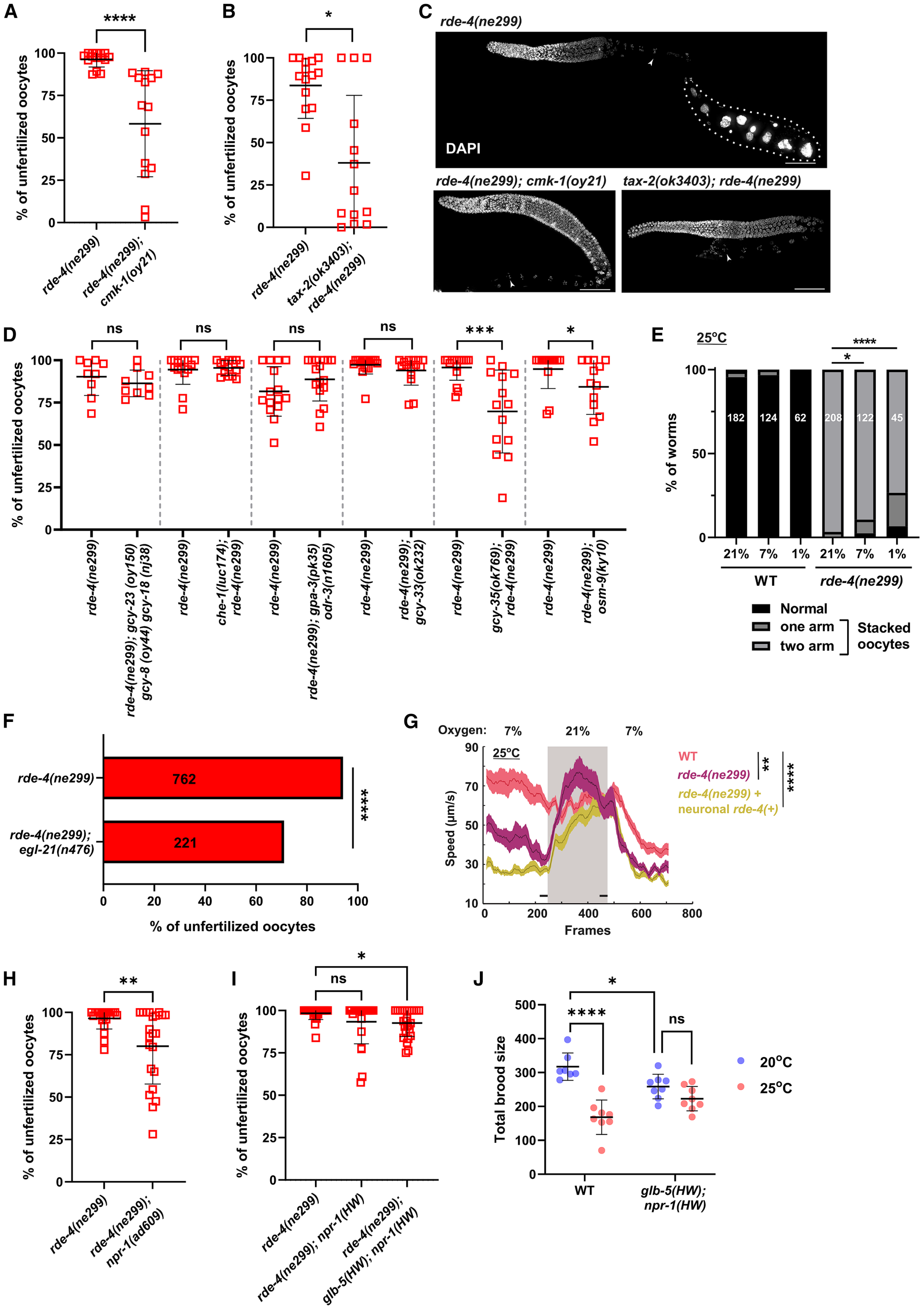

Figure 3. Oxygen-sensing neurons inhibit reproduction

(A–C) Broad inhibition of sensory perception by knocking out *cmk-1* or *tax-2* rescues the fertility defects of *rde-4(ne299)* at 25°C. (C) Endomitotic oocytes in *rde-4(ne299)* are highlighted. Arrows indicate the −1 oocytes, located closest to the spermatheca. DAPI staining was done in day-1 adults. Scale bars, 50 µm.

(legend continued on next page)

in developmental timing.[67] Using this approach, we found that genes downregulated in *rde-4(–)* mutants at 25°C compared with those at 20°C are significantly enriched for mitochondrial processes (Figure S3C). We observed a similar pattern of mitochondrial gene downregulation when comparing *rde-4(–)* mutants to wild-type animals at 25°C (Figure S3D).

We found that deletion of *gcy-33*, an oxygen receptor gene expressed in BAG neurons,[64,68] did not affect the fertility of *rde-4(–)* mutants (Figure 3D). In contrast, deleting *gcy-35*, which encodes a heme-containing oxygen receptor expressed in the URX, AQR, PQR, SDQ, ALN, and PLN neurons that promote hyperoxia avoidance, rescues sterility of *rde-4(–)* mutants (unfertilized oocytes: 95.7% in *rde-4(ne299)* versus 69.8% in *gcy-35(ok769);rde-4(ne299)*) (Figures 3D and S3E–S3G). To confirm these results, we generated an in-house loss-of-function allele in *gcy-35* using CRISPR-Cas9 and observed similar effects (unfertilized oocytes: 95.3% in *rde-4(ne299)* versus 73.3% in *gcy-35(db2020);rde-4(ne299)*) (Figures S3G–S3J). We further showed that genetically ablating URX, AQR, and PQR neurons alone by expressing the cell-death activator gene *egl-1* driven by the *gcy-36* promoter (*qaIs2241*) is insufficient to rescue *rde-4(–)* sterility (Figure S3K). This is consistent with a previous study showing that animals carrying *qaIs2241* transgene retain hyperoxia avoidance, unlike *gcy-35(–)* mutants,[62] and points to other *gcy-35*-expressing neurons, including SDQ, ALN, and PLN, as potential candidates for regulating germline development in *rde-4(–)* animals. Given that these neurons do not appear to express TAX-2,[54] additional sensory neurons are likely to contribute to the regulation of germline development.

TRPV sensory channel OSM-9 acts in the polymodal nociceptive ASH neurons to regulate avoidance of high osmolarity, chemical repellents, and touch, and also acts in non-nociceptive cell types to mediate olfactory responses and sensory adaptation.[69–71] Of note, OSM-9 has been shown to function in ASH and serotonergic ADF neurons to promote hyperoxia avoidance.[62,72] We found that knocking out *osm-9* modestly rescues *rde-4(–)* phenotype (unfertilized oocytes: 94.7% in *rde-4(ne299)* versus 84.3% in *rde-4(ne299);osm-9(ky10)*) (Figure 3D). However, removing the tryptophan hydroxylase enzyme required for serotonin biosynthesis, TPH-1, does not affect *rde-4(–)* fertility (Figure S3L), nor does genetic ablation of ASH neurons alone (Figure S3M). Together, these results indicate that multiple oxygen-sensing neurons function synergistically to regulate sRNA-mediated germline development.

Hypoxia exposure, for example, living at high altitude, has been known to negatively impact male fertility in animals.[73] Here, we show that mere perception of oxygen—without changing oxygen levels—can antagonize sperm development and fertility modulated by sRNA pathways. We propose that inhibiting oxygen-sensing neurons may mimic cues associated with active bacterial growth that create oxygen sinks, suggestive of conditions favorable for reproduction in *C. elegans*. Supporting this notion, exposure to low oxygen levels (7% and 1%) partially alleviates the germline defects of *rde-4(–)* mutants at 25°C (Figure 3E). Note that, in our standard assay, day-1 adults were transferred to fresh plates for egg laying (see STAR Methods). To avoid confounding effects of shifting animals from low oxygen to normoxia, we instead incubated worms at 25°C for 24 h without transfers and scored germline morphological defects using the germline marker *mex-5::gfp*.

Neuronal signaling pathways, especially the sensory system, exhibit extensive evolutionary plasticity and may be subjected to positive selection in response to changing habitats.[74,75] In the canonical *C. elegans* "wild-type" strain (N2), laboratory domestication led to the fixation of a gain-of-function allele in the neuropeptide Y homolog *npr-1* and a duplication/insertion in hexacoordinated globin gene *glb-5*, both of which alter the animals' response to oxygen and carbon dioxide.[63,76,77] These laboratory-derived alleles are absent in LSJ1, a sister strain derived from N2 at least six years before its cryopreservation in 1969.[78]

In N2, the gain-of-function NPR-1(215V) downregulates the activities of oxygen-sensing AQR, PQR, and URX neurons, thereby suppressing hyperoxia avoidance. NPR-1(215V) animals do not respond to drops in oxygen levels and are solitary feeders, dispersing across the bacterial lawn. In contrast, wild isolates such as CB4856 from Hawaii (HW), which carry NPR-1(215F), or animals with an *npr-1* loss-of-function allele, slow their movement in response to reduced oxygen and exhibit social feeding (Figure S3N), aggregating along the bacterial lawn border where oxygen levels are lower (effective oxygen concentration of ~12.8% versus ~17.1% in the center of the lawn).[63] Given the central role of neuropeptide signaling in *C. elegans* oxygen sensing, we asked whether it also influences germline development in *rde-4(–)* mutants. Removing carboxypeptidase E ortholog EGL-21, which is required to process endogenous neuropeptides, partially restores *rde-4(–)* fertility (unfertilized oocytes: 94.2% in *rde-4(ne299)* versus 71.0% in *rde-4(ne299);egl-21(n476)*) (Figure 3F). We observed a similar effect by depleting UNC-31/CAPS[79] required for neuropeptide release

(D) Inhibiting oxygen perception, but not the other sensory modalities, improves *rde-4(ne299)* fertility.
(E) Low oxygen levels (7% and 1%) partially rescue germline defects in *rde-4(ne299)* at 25°C. L4 animals were transferred to a glove box maintained at the desired oxygen level, and the presence of stacked unfertilized oocytes in one (one arm) or both (two arms) gonadal arms in day-1 adults was quantified.
(F) Blocking neuropeptide processing rescues *rde-4(ne299)* sterility. Note that *egl-2(n476)* has egg-laying defects independent of fertility.
(G) *rde-4(ne299)* and *pigSi3[Psng-1::rde-4]; rde-4(ne299)* mutants show increased responses to 7% and 21% oxygen at 25°C, compared with wild type. $n = 7–9$ assays, 20–25 animals per assay. Solid lines indicate average speed, and error indicates SEM. Black horizontal bars indicate time intervals used for statistical tests.
(H) *npr-1(ad609)* mutation rescues *rde-4(ne299)* sterility.
(I and J) Ancestral (HW) alleles of *npr-1* and *glb-5* partially restore *rde-4(ne299)* fertility. *glb-5(HW);npr-1(HW)* mutants do not show heat-induced loss of fertility. Error represents mean ± SD.
For (A), (B), (D), (G), and (H–J), statistical significance was determined by Mann-Whitney tests. Multiple comparison corrections were applied where appropriate. For (E) and (F), the numbers indicate the total number of animals scored, and statistical significance was determined by Fisher's exact test. Not significant, $p > 0.05$; *$p \leq 0.05$; **$p < 0.01$; p*** < 0.001; ****$p < 0.0001$.
See also Figure S3.

from dense-core vesicles, using the auxin-inducible degron (AID) system (unfertilized oocytes: 88.3% in EtOH versus 70.3% in 1 mM auxin) (Figure S3O). We subsequently screened a subset of FMRFamide-like neuropeptides previously implicated in oxygen response and found that loss of FLP-8, FLP-19, or FLP-21 failed to restore fertility in *rde-4(−)* mutants (Figures S3P–S3R), suggesting the involvement of other neuropeptides that we did not test here.

At 20°C in the presence of food, *rde-4(ne299)* mutants behave similarly to wild-type N2 in their oxygen response (Figure S3S). However, at 25°C, we found that *rde-4(ne299)* mutants, unlike N2, respond to oxygen, slowing their movement at 7% and speeding up at 21% oxygen (Figure 3G). This phenotype is alleviated by loss of *gcy-35* (Figure S3T). Unexpectedly, pan-neuronal overexpression of *rde-4* does not rescue the mutant oxygen response (Figure 3G), suggesting that RNAi may play complex, dose-sensitive roles in the nervous system. Moreover, neuronal *rde-4(+)* (*pigSi3* transgene) may regulate germline development independent of the oxygen-sensing neural pathway, which calls for future studies.

Interestingly, we found that strong loss-of-function mutations in *npr-1* rescue the fertility defects of *rde-4(−)* mutants (unfertilized oocytes: 96.5% in *rde-4(ne299)* versus 80.0% in *rde-4(ne299);npr-1(ad609)*) (Figure 3H). We confirmed these results using two independent EMS-induced alleles (*ok1447* and *ad609*) (unfertilized oocytes: 95.6% in *rde-4(ne299)* versus 78.3% in *rde-4(ne299);npr-1(ok1447)*) (Figures S3U and S3V). To test whether the NPR-1 regulation of germline thermotolerance in *rde-4(−)* mutants is linked to social behaviors, we repeated the experiments on uniform bacterial lawns, which prevent oxygen gradients arising from uneven bacterial thickness on standard lawns. We similarly observed rescue under these conditions (Figure S4W), indicating that NPR-1 exerts pleiotropic effects on life-history traits independent of foraging behaviors, perhaps by altering metabolism, as previously shown.[80] Although *npr-1(HW)* alone does not affect *rde-4(−)* fertility, *glb-5(HW);npr-1(HW)* double mutant weakly but significantly rescues *rde-4(−)* phenotype (unfertilized oocytes: 98.4% in *rde-4(ne299)* versus 92.6% in *rde-4(ne299);glb-5(HW);npr-1(HW)*) (Figure 3I). Consistent with previous findings that these two laboratory-derived alleles confer fitness advantages in a standard laboratory environment, *glb-5(HW);npr-1(HW)* mutants show a lower brood size compared with wild type at 20°C (Figure 3J).[80,81] At 25°C, the brood size of wild-type animals drops significantly owing to sperm dysfunction[82]; we found that this decline is not evident in *glb-5(HW);npr-1(HW)* (Figure 3J), suggesting that the ancestral alleles of *npr-1* and *glb-5* promote reproductive resilience under heat stress—a major challenge in their natural habitat.

Hence, our results indicate that laboratory domestication alters the oxygen-sensing circuit, which subsequently impacts germline development under heat stress. However, we note that knocking out *rde-4* in LSJ1 and CB4856 causes severe loss of fertility, as observed in N2 (Figure S3X), suggesting other natural genetic variants in these strains may act to affect germline development and/or sRNA pathways.[83–86] Indeed, AGO genes and sRNA pathways are evolving rapidly, exhibiting extensive intraspecies variation that may have broad impacts on germline gene regulation.[84,87] Additionally, other laboratory-derived alleles have been shown to affect sperm development,[85,86] including a deletion in *nurf-1*, which encodes the ortholog of the BPTF subunit of the NURF chromatin remodeling complex, that arose in the LSJ1 lineage.[86]

Neuronal signaling influences genome stability in the germline

We note that, although mating largely rescues the fertility defect in *rde-4(−)* mutants, it does not fully restore brood size to wild-type levels, suggesting additional, non-sperm-origin defects (Figure 4A). To investigate further, we examined RAD-51/RecA, which localizes to the double-strand break (DSB) repair loci, in the oogenic germline of day-1 hermaphrodites.[88,89] In wild-type gonads, RAD-51 appears as distinct foci mostly in the leptotene/zygotene and pachytene stages, which coincide with the onset of meiosis (Figures 4B and 4C). In contrast, at 25°C, but not at 20°C, *rde-4(−)* mutants exhibited an elevated frequency of DSBs throughout the germline, including the mitotic proliferative stem cells, indicating compromised genome integrity under heat stress (Figures 4D and S4A). Remarkably, consistent with our earlier findings, this defect was rescued by neuronal expression of *rde-4(+)* (Figures 4E–4G). Moreover, eliminating *cmk-1* or *tax-2* reduces the number of RAD-51 foci in *rde-4(−)* mutants (Figures 4H, 4J, 4K, and S4B). Finally, loss of *gcy-35* or *egl-21* rescued the DSBs in *rde-4(−)* (Figures 4I–4M). Our data do not preclude the possibility that DNA damage machinery in these mutants responds to altered sperm signals.[90] Nevertheless, these results support the conclusion that neuronal activity—particularly the oxygen-sensing circuit—plays an important role in regulating reproductive robustness. Perception of oxygen levels may modulate germline maintenance as low oxygen levels correlate with bacteria-rich environments and a reproductive permissive niche.

DISCUSSION

One of the key features of sRNAs/AGOs is their capacity to help animals mitigate environmental changes by rapidly reprogramming gene expression. This is evident in their conserved roles in conferring stress tolerance to gametes from plants to mammals.[3,91,92] In *C. elegans*, previous studies have shown that ERI-1/3/5, RRF-3, and DCR-1, together with downstream ALG-3/4 in the endo-siRNA pathway, are important for sperm development under heat stress[12,16]; however, the role of RDE-4 in this pathway has not been explicitly addressed. Here, we found that RDE-4 is crucial for both spermatogenic and oogenic germline thermotolerance. Loss of RDE-4 at elevated temperature leads to misregulation of spermatogenic genes, chromosomal defects in the sperm, and an increased DSB in oogenic germ cells, highlighting the crucial role of endo-siRNAs in maintaining reproductive robustness in stressful conditions.

Regulatory sRNAs can exert cell non-autonomous effects via microvesicles or exosomes, influencing diverse biological processes in both health and disease.[93,94] The intercellular transfer of RNAs and other molecular cargoes mediates soma-germline interactions from plants to mammals, regulating germline gene expression.[18,20,21] In this study, we showed that neuronal RNAi modulates germline development in response to genetic and environmental perturbations, buffering developmental systems

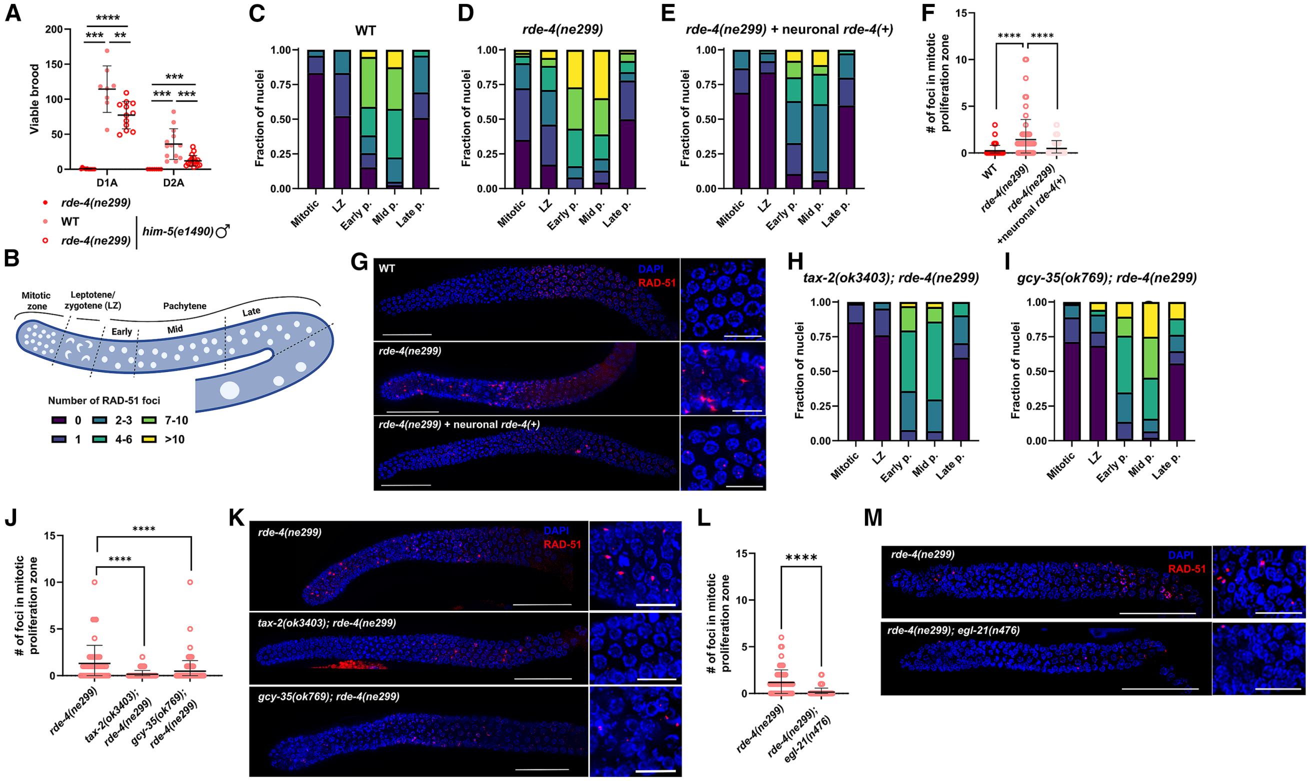

Figure 4. Neuronal RDE-4 and impaired oxygen-sensing neural pathways promote germline genome stability

(A) Crossing *rde-4(ne299)* hermaphrodites to *him-5(e1490)* males does not completely restore brood size at 25°C. D1A: day-1 adults (overnight mating initiated at the L4 stage); D2A: day-2 adults (overnight mating initiated in day-1 adults).

(B–G) Neuronal *rde-4(+)* rescues DSBs in *rde-4(ne299)* at 25°C. L4 animals were transferred from 20°C to 25°C for ~24 h. Antibody staining was performed on day-1 adults.

(H–M) Loss of oxygen sensation (*tax-2(ok3403)* or *gcy-35(ok769)*) or neuropeptide signaling (*egl-21(n476)*) rescues DSBs in *rde-4(ne299)*.

Images in (G), (K), and (M) show whole gonads stained with RAD-51 (red) and DAPI (blue). Scale bars, 50 μm. The right panels show magnification of mitotic nuclei. Scale bars, 10 μm.

For all relevant panels, error represents mean ± SD. $^{**}p < 0.01$; $^{***}p < 0.001$; $^{****}p < 0.0001$ by Mann-Whitney tests. Multiple comparison corrections were applied where appropriate.

See also Figure S4.

under stress. This may allow distributed robustness—a common feature of metabolic and developmental programs.[95,96] Although more work is required to define the detailed mechanism underlying neuron-to-germline communication during heat stress, our data showed that neuronal sRNAs can regulate reproduction through a SID-1-independent mechanism. Other candidate mediators of RNA transfer from neurons to the germline include RSD (RNA spreading defective) proteins, which have been implicated in systemic RNAi in *C. elegans*,[97] as well as gap junctions,[98,99] warranting future investigation. It also remains possible that other tissues—for example, the intestine, which can influence germline epigenome via yolk provisioning[100,101]—contribute to amplifying and passing on epigenetic information from neurons to germline.

Remarkably, we provided evidence that sensory signaling may affect germline development during stress. Specifically, disrupting GCY-35, a soluble guanylate cyclase involved in neuronal oxygen sensing and neuropeptide signaling, promotes germline maintenance and reproduction in RNAi mutants at high temperature, even without altering ambient oxygen levels. Serendipitously, we uncovered that RNAi may suppress oxygen response at high temperature in N2, either by modulating oxygen sensory perception directly or by more broadly affecting neuronal development and functions. This can be further studied by conditionally depleting *rde-4* in a defined window to avoid potential developmental defects in constitutive mutants and examining neuronal differentiation states using the NeuroPAL system.[102] Although we cannot exclude the possibility that impairing the oxygen-sensing circuit elicits a systemic stress response that indirectly affects germline physiology, or that GCY-35 may have non-canonical roles outside of direct oxygen sensing, our study strongly supports a model in which signaling *initiated* in sensory neurons can exert non-cell-autonomous effects on the germline developmental program. This neuron-to-germline interaction may, in some cases, trigger a lasting transgenerational epigenetic memory.[56] Hence, we propose that sRNAs integrate sensory information, promote developmental plasticity and reproductive adaptation, and potentially facilitate epigenetic buffering in fluctuating environments.[103–106]

CellPress
OPEN ACCESS

RESOURCE AVAILABILITY

Lead contact

Further information and requests for resources and reagents should be directed to and will be fulfilled by the lead contact, Oded Rechavi (odedrechavi@gmail.com).

Materials availability

C. elegans strains generated in this study are available upon request.

Data and code availability

- All NGS data are available through GEO under accession number GSE331410.
- This paper does not report original code.
- Any additional information required to reanalyze the data reported in this paper is available from the lead contact upon request.

ACKNOWLEDGMENTS

We thank Itai Reiger for their assistance with experiments. We thank Cori Bargmann (Rockefeller University) for providing introgressed strains carrying HW alleles of *npr-1* and *glb-5*. Some graphics were created with Biorender.com. We are grateful to WormBase for providing valuable data and resources. Some strains were provided by the Caenorhabditis Genetics Center (CGC), which is funded by NIH Office of Research Infrastructure Programs (P40 OD010440). O.R. is grateful for the support of the Morris Kahn Foundation. C.K.E. was supported by an EMBO fellowship ALTF 6-2022. This work is funded by Eric and Wendy Schmidt Fund for Strategic Innovation Polymath Award 0140001000 (O.R.); European Research Council grant 335624 (O.R.); Israel Science Foundation 979/21 (Y.B.T.); and the US-Israel Binational Science Foundation 2023036 (Y.B.T.).

AUTHOR CONTRIBUTIONS

Conceptualization, C.K.E. and O.R.; methodology, C.K.E. and H.A.; investigation, C.K.E., H.A., S.W., A.M., H.S., L.K., M.V., G.T., S.A., H.G., R.P., O.A., and R.B.; visualization, C.K.E., H.A., and H.S.; funding acquisition, Y.B.T. and O.R.; supervision, C.K.E., M.d.B., Y.B.T., and O.R.; writing—original draft, C.K.E.; writing—review & editing, C.K.E., H.A., H.S., Y.B.T., and O.R.

DECLARATION OF INTERESTS

The authors declare no competing interests.

STAR★METHODS

Detailed methods are provided in the online version of this paper and include the following:

- KEY RESOURCES TABLE
- EXPERIMENTAL MODEL AND STUDY PARTICIPANT DETAILS
- METHOD DETAILS
  - Fertility assay
  - Fertility assay on uniform bacterial lawn
  - RNAi
  - AID-Mediated degradation of UNC-31
  - RNA isolation
  - RNA-seq
  - Bioinformatics
  - CRISPR/Cas9-mediated knockout of *rde-4*
  - CRISPR/Cas9-mediated knockout of *gcy-35*
  - Low oxygen exposure experiment
  - Locomotion assays
  - Antibody and DAPI staining
- QUANTIFICATION AND STATISTICAL ANALYSIS

SUPPLEMENTAL INFORMATION

Supplemental information can be found online at https://doi.org/10.1016/j.cub.2026.06.016.

Received: October 7, 2025
Revised: April 17, 2026
Accepted: June 8, 2026
Published: July 6, 2026

REFERENCES

1. Walsh, B.S., Parratt, S.R., Hoffmann, A.A., Atkinson, D., Snook, R.R., Bretman, A., and Price, T.A.R. (2019). The Impact of Climate Change on Fertility. Trends Ecol. Evol. *34*, 249–259. https://doi.org/10.1016/j.tree.2018.12.002.
2. De Jaeger-Braet, J., and Schnittger, A. (2024). Heating up meiosis - Chromosome recombination and segregation under high temperatures. Curr. Opin. Plant Biol. *80*, 102548. https://doi.org/10.1016/j.pbi.2024.102548.
3. Bourc'his, D., and Voinnet, O. (2010). A Small-RNA Perspective on Gametogenesis, Fertilization, and Early Zygotic Development. Science *330*, 617–622. https://doi.org/10.1126/science.1194776.
4. Cox, D.N., Chao, A., Baker, J., Chang, L., Qiao, D., and Lin, H. (1998). A novel class of evolutionarily conserved genes defined by piwi are essential for stem cell self-renewal. Genes Dev. *12*, 3715–3727. https://doi.org/10.1101/gad.12.23.3715.
5. Aravin, A.A., Sachidanandam, R., Girard, A., Fejes-Toth, K., and Hannon, G.J. (2007). Developmentally regulated piRNA clusters implicate MILI in transposon control. Science *316*, 744–747. https://doi.org/10.1126/science.1142612.
6. Brennecke, J., Aravin, A.A., Stark, A., Dus, M., Kellis, M., Sachidanandam, R., and Hannon, G.J. (2007). Discrete small RNA-generating loci as master regulators of transposon activity in Drosophila. Cell *128*, 1089–1103. https://doi.org/10.1016/j.cell.2007.01.043.
7. Carmell, M.A., Girard, A., van de Kant, H.J.G., Bourc'his, D., Bestor, T.H., de Rooij, D.G., and Hannon, G.J. (2007). MIWI2 is essential for spermatogenesis and repression of transposons in the mouse male germline. Dev. Cell *12*, 503–514. https://doi.org/10.1016/j.devcel.2007.03.001.
8. Hilz, S., Modzelewski, A.J., Cohen, P.E., and Grimson, A. (2016). The roles of microRNAs and siRNAs in mammalian spermatogenesis. Development *143*, 3061–3073. https://doi.org/10.1242/dev.136721.
9. Stein, P., Rozhkov, N.V., Li, F., Cárdenas, F.L., Davydenk, O., Vandivier, L.E., Gregory, B.D., Hannon, G.J., and Schultz, R.M. (2015). Essential Role for Endogenous siRNAs during Meiosis in Mouse Oocytes. PLOS Genet. *11*, e1005013. https://doi.org/10.1371/journal.pgen.1005013.
10. Taborska, E., Pasulka, J., Malik, R., Horvat, F., Jenickova, I., Jelić Matošević, Z.J., and Svoboda, P. (2019). Restricted and non-essential redundancy of RNAi and piRNA pathways in mouse oocytes. PLOS Genet. *15*, e1008261. https://doi.org/10.1371/journal.pgen.1008261.
11. Vrettos, N., Oppelt, J., Zoch, A., Sgourdou, P., Yoshida, H., Song, B., Fink, R., O'Carroll, D., and Mourelatos, Z. (2024). MIWI N-terminal arginines orchestrate generation of functional pachytene piRNAs and spermiogenesis. Nucleic Acids Res. *52*, 6558–6570. https://doi.org/10.1093/nar/gkae193.
12. Conine, C.C., Batista, P.J., Gu, W., Claycomb, J.M., Chaves, D.A., Shirayama, M., and Mello, C.C. (2010). Argonautes ALG-3 and ALG-4 are required for spermatogenesis-specific 26G-RNAs and thermotolerant sperm in Caenorhabditis elegans. Proc. Natl. Acad. Sci. USA *107*, 3588–3593. https://doi.org/10.1073/pnas.0911685107.
13. Charlesworth, A.G., Seroussi, U., Lehrbach, N.J., Renaud, M.S., Sundby, A.E., Molnar, R.I., Lao, R.X., Willis, A.R., Woock, J.R., Aber, M.J., et al. (2021). Two isoforms of the essential C. elegans Argonaute CSR-1

differentially regulate sperm and oocyte fertility. Nucleic Acids Res. *49*, 8836–8865. https://doi.org/10.1093/nar/gkab619.

14. Batista, P.J., Ruby, J.G., Claycomb, J.M., Chiang, R., Fahlgren, N., Kasschau, K.D., Chaves, D.A., Gu, W., Vasale, J.J., Duan, S., et al. (2008). PRG-1 and 21U-RNAs interact to form the piRNA complex required for fertility in C. elegans. Mol. Cell *31*, 67–78. https://doi.org/10.1016/j.molcel.2008.06.002.
15. Buckley, B.A., Burkhart, K.B., Gu, S.G., Spracklin, G., Kershner, A., Fritz, H., Kimble, J., Fire, A., and Kennedy, S. (2012). A nuclear Argonaute promotes multigenerational epigenetic inheritance and germline immortality. Nature *489*, 447–451. https://doi.org/10.1038/nature11352.
16. Gent, J.I., Schvarzstein, M., Villeneuve, A.M., Gu, S.G., Jantsch, V., Fire, A.Z., and Baudrimont, A. (2009). A Caenorhabditis elegans RNA-directed RNA polymerase in sperm development and endogenous RNA interference. Genetics *183*, 1297–1314. https://doi.org/10.1534/genetics.109.109686.
17. Pavelec, D.M., Lachowiec, J., Duchaine, T.F., Smith, H.E., and Kennedy, S. (2009). Requirement for the ERI/DICER Complex in Endogenous RNA Interference and Sperm Development in Caenorhabditis elegans. Genetics *183*, 1283–1295. https://doi.org/10.1534/genetics.109.108134.
18. Conine, C.C., and Rando, O.J. (2022). Soma-to-germline RNA communication. Nat. Rev. Genet. *23*, 73–88. https://doi.org/10.1038/s41576-021-00412-1.
19. Corrigan, L., Redhai, S., Leiblich, A., Fan, S.-J., Perera, S.M.W., Patel, R., Gandy, C., Wainwright, S.M., Morris, J.F., Hamdy, F., et al. (2014). BMP-regulated exosomes from Drosophila male reproductive glands reprogram female behavior. J. Cell Biol. *206*, 671–688. https://doi.org/10.1083/jcb.201401072.
20. Sharma, U., Conine, C.C., Shea, J.M., Boskovic, A., Derr, A.G., Bing, X.Y., Belleannee, C., Kucukural, A., Serra, R.W., Sun, F., et al. (2016). Biogenesis and function of tRNA fragments during sperm maturation and fertilization in mammals. Science *351*, 391–396. https://doi.org/10.1126/science.aad6780.
21. Chen, Q., Yan, M., Cao, Z., Li, X., Zhang, Y., Shi, J., Feng, G., Peng, H., Zhang, X., Zhang, Y., et al. (2016). Sperm tsRNAs contribute to intergenerational inheritance of an acquired metabolic disorder. Science *351*, 397–400. https://doi.org/10.1126/science.aad7977.
22. Rompala, G.R., Mounier, A., Wolfe, C.M., Lin, Q., Lefterov, I., and Homanics, G.E. (2018). Heavy Chronic Intermittent Ethanol Exposure Alters Small Noncoding RNAs in Mouse Sperm and Epididymosomes. Front. Genet. *9*, 32. https://doi.org/10.3389/fgene.2018.00032.
23. Rompala, G.R., Ferguson, C., and Homanics, G.E. (2020). Coincubation of sperm with epididymal extracellular vesicle preparations from chronic intermittent ethanol-treated mice is sufficient to impart anxiety-like and ethanol-induced behaviors to adult progeny. Alcohol *87*, 111–120. https://doi.org/10.1016/j.alcohol.2020.05.001.
24. Chendrimada, T.P., Gregory, R.I., Kumaraswamy, E., Norman, J., Cooch, N., Nishikura, K., and Shiekhattar, R. (2005). TRBP recruits the Dicer complex to Ago2 for microRNA processing and gene silencing. Nature *436*, 740–744. https://doi.org/10.1038/nature03868.
25. Czech, B., Malone, C.D., Zhou, R., Stark, A., Schlingeheyde, C., Dus, M., Perrimon, N., Kellis, M., Wohlschlegel, J.A., Sachidanandam, R., et al. (2008). An endogenous small interfering RNA pathway in Drosophila. Nature *453*, 798–802. https://doi.org/10.1038/nature07007.
26. Liu, Q., Rand, T.A., Kalidas, S., Du, F., Kim, H.-E., Smith, D.P., and Wang, X. (2003). R2D2, a bridge between the initiation and effector steps of the Drosophila RNAi pathway. Science *301*, 1921–1925. https://doi.org/10.1126/science.1088710.
27. Tabara, H., Sarkissian, M., Kelly, W.G., Fleenor, J., Grishok, A., Timmons, L., Fire, A., and Mello, C.C. (1999). The rde-1 gene, RNA interference, and transposon silencing in C. elegans. Cell *99*, 123–132. https://doi.org/10.1016/s0092-8674(00)81644-x.
28. Tabara, H., Yigit, E., Siomi, H., and Mello, C.C. (2002). The dsRNA Binding Protein RDE-4 Interacts with RDE-1, DCR-1, and a DExH-Box Helicase to Direct RNAi in C. elegans. Cell *109*, 861–871. https://doi.org/10.1016/S0092-8674(02)00793-6.
29. Parker, G.S., Maity, T.S., and Bass, B.L. (2008). dsRNA binding properties of RDE-4 and TRBP reflect their distinct roles in RNAi. J. Mol. Biol. *384*, 967–979. https://doi.org/10.1016/j.jmb.2008.10.002.
30. Parker, G.S., Eckert, D.M., and Bass, B.L. (2006). RDE-4 preferentially binds long dsRNA and its dimerization is necessary for cleavage of dsRNA to siRNA. RNA *12*, 807–818. https://doi.org/10.1261/rna.2338706.
31. Welker, N.C., Pavelec, D.M., Nix, D.A., Duchaine, T.F., Kennedy, S., and Bass, B.L. (2010). Dicer's helicase domain is required for accumulation of some, but not all, C. elegans endogenous siRNAs. RNA *16*, 893–903. https://doi.org/10.1261/rna.2122010.
32. Knittel, T.L., Montgomery, B.E., Sprister, R.A., Magelky, C.N., Smith, M.J., Soto-Ojeda, M., Guthrie, M., Phillips, C.M., and Montgomery, T.A. (2025). Argonaute-siRNA loading via the RNA-binding protein RDE-4 in *C. elegans*. Curr. Biol. *35*, 5897–5907.e6. https://doi.org/10.1016/j.cub.2025.10.042.
33. Blanchard, D., Parameswaran, P., Lopez-Molina, J., Gent, J., Saynuk, J.F., and Fire, A. (2011). On the nature of in vivo requirements for rde-4 in RNAi and developmental pathways in C. elegans. RNA Biol. *8*, 458–467. https://doi.org/10.4161/rna.8.3.14657.
34. Ward, S., and Carrel, J.S. (1979). Fertilization and sperm competition in the nematode Caenorhabditis elegans. Dev. Biol. *73*, 304–321. https://doi.org/10.1016/0012-1606(79)90069-1.
35. Meneely, P.M., McGovern, O.L., Heinis, F.I., and Yanowitz, J.L. (2012). Crossover Distribution and Frequency Are Regulated by him-5 in Caenorhabditis elegans. Genetics *190*, 1251–1266. https://doi.org/10.1534/genetics.111.137463.
36. Ketting, R.F., and Cochella, L. (2021). Concepts and functions of small RNA pathways in C. elegans. Curr. Top. Dev. Biol. *144*, 45–89. https://doi.org/10.1016/bs.ctdb.2020.08.002.
37. Seroussi, U., Lugowski, A., Wadi, L., Lao, R.X., Willis, A.R., Zhao, W., Sundby, A.E., Charlesworth, A.G., Reinke, A.W., and Claycomb, J.M. (2023). A comprehensive survey of C. elegans argonaute proteins reveals organism-wide gene regulatory networks and functions. eLife *12*, e83853. https://doi.org/10.7554/eLife.83853.
38. Yigit, E., Batista, P.J., Bei, Y., Pang, K.M., Chen, C.-C.G., Tolia, N.H., Joshua-Tor, L., Mitani, S., Simard, M.J., and Mello, C.C. (2006). Analysis of the C. elegans Argonaute Family Reveals that Distinct Argonautes Act Sequentially during RNAi. Cell *127*, 747–757. https://doi.org/10.1016/j.cell.2006.09.033.
39. Vasale, J.J., Gu, W., Thivierge, C., Batista, P.J., Claycomb, J.M., Youngman, E.M., Duchaine, T.F., Mello, C.C., and Conte, D. (2010). Sequential rounds of RNA-dependent RNA transcription drive endogenous small-RNA biogenesis in the ERGO-1/Argonaute pathway. Proc. Natl. Acad. Sci. USA *107*, 3582–3587. https://doi.org/10.1073/pnas.0911908107.
40. Han, T., Manoharan, A.P., Harkins, T.T., Bouffard, P., Fitzpatrick, C., Chu, D.S., Thierry-Mieg, D., Thierry-Mieg, J., and Kim, J.K. (2009). 26G endo-siRNAs regulate spermatogenic and zygotic gene expression in Caenorhabditis elegans. Proc. Natl. Acad. Sci. USA *106*, 18674–18679. https://doi.org/10.1073/pnas.0906378106.
41. Zhang, C., Montgomery, T.A., Gabel, H.W., Fischer, S.E.J., Phillips, C.M., Fahlgren, N., Sullivan, C.M., Carrington, J.C., and Ruvkun, G. (2011). mut-16 and other mutator class genes modulate 22G and 26G siRNA pathways in Caenorhabditis elegans. Proc. Natl. Acad. Sci. USA *108*, 1201–1208. https://doi.org/10.1073/pnas.1018695108.
42. Duchaine, T.F., Wohlschlegel, J.A., Kennedy, S., Bei, Y., Conte, D., Pang, K., Brownell, D.R., Harding, S., Mitani, S., Ruvkun, G., et al. (2006). Functional Proteomics Reveals the Biochemical Niche of C. elegans DCR-1 in Multiple Small-RNA-Mediated Pathways. Cell *124*, 343–354. https://doi.org/10.1016/j.cell.2005.11.036.
43. Posner, R., Toker, I.A., Antonova, O., Star, E., Anava, S., Azmon, E., Hendricks, M., Bracha, S., Gingold, H., and Rechavi, O. (2019).

CellPress
OPEN ACCESS

Neuronal Small RNAs Control Behavior Transgenerationally. Cell *177*, 1814–1826.e15. https://doi.org/10.1016/j.cell.2019.04.029.

44. Conine, C.C., Moresco, J.J., Gu, W., Shirayama, M., Conte, D., Yates, J.R., and Mello, C.C. (2013). Argonautes promote male fertility and provide a paternal memory of germline gene expression in C. elegans. Cell *155*, 1532–1544. https://doi.org/10.1016/j.cell.2013.11.032.

45. Winston, W.M., Molodowitch, C., and Hunter, C.P. (2002). Systemic RNAi in C. elegans requires the putative transmembrane protein SID-1. Science *295*, 2456–2459. https://doi.org/10.1126/science.1068836.

46. Artiles, K.L., Fire, A.Z., and Frøkjær-Jensen, C. (2019). Assessment and maintenance of unigametic germline inheritance for C. elegans. Dev. Cell *48*, 827–839.e9. https://doi.org/10.1016/j.devcel.2019.01.020.

47. Besseling, J., and Bringmann, H. (2016). Engineered non-Mendelian inheritance of entire parental genomes in C. elegans. Nat. Biotechnol. *34*, 982–986. https://doi.org/10.1038/nbt.3643.

48. Sulston, J.E., Schierenberg, E., White, J.G., and Thomson, J.N. (1983). The embryonic cell lineage of the nematode Caenorhabditis elegans. Dev. Biol. *100*, 64–119. https://doi.org/10.1016/0012-1606(83)90201-4.

49. Hubbard, E.J.A. (2014). FLP/FRT and Cre/*lox* recombination technology in *C. elegans*. Methods *68*, 417–424. https://doi.org/10.1016/j.ymeth.2014.05.007.

50. Bharadwaj, P.S., and Hall, S.E. (2017). Endogenous RNAi Pathways Are Required in Neurons for Dauer Formation in Caenorhabditis elegans. Genetics *205*, 1503–1516. https://doi.org/10.1534/genetics.116.195438.

51. Tonkin, L.A., and Bass, B.L. (2003). Mutations in RNAi Rescue Aberrant Chemotaxis of ADAR Mutants. Science *302*, 1725. https://doi.org/10.1126/science.1091340.

52. Juang, B.-T., Gu, C., Starnes, L., Palladino, F., Goga, A., Kennedy, S., and L'Etoile, N.D. (2013). Endogenous Nuclear RNAi Mediates Behavioral Adaptation to Odor. Cell *154*, 1010–1022. https://doi.org/10.1016/j.cell.2013.08.006.

53. Kennedy, L.M., and Grishok, A. (2014). Neuronal migration is regulated by endogenous RNAi and chromatin-binding factor ZFP-1/AF10 in Caenorhabditis elegans. Genetics *197*, 207–220. https://doi.org/10.1534/genetics.114.162917.

54. Coburn, C.M., and Bargmann, C.I. (1996). A Putative Cyclic Nucleotide–Gated Channel Is Required for Sensory Development and Function in C. elegans. Neuron *17*, 695–706. https://doi.org/10.1016/S0896-6273(00)80201-9.

55. Inada, H., Ito, H., Satterlee, J., SenGupta, P., Matsumoto, K., and Mori, I. (2006). Identification of Guanylyl Cyclases That Function in Thermosensory Neurons of Caenorhabditis elegans. Genetics *172*, 2239–2252. https://doi.org/10.1534/genetics.105.050013.

56. Teichman, G., Sela, M., Ewe, C.K., Rieger, I., Anava, S., Mor, Y., Szántó, P., Meyer, D.H., Doron, H., Shachar, O., et al. (2024). Perception of Temperature Even in the Absence of Actual Change is Sufficient to Drive Transgenerational Epigenetic Inheritance. Preprint at bioRxiv. https://doi.org/10.1101/2024.12.02.626416.

57. Uchida, O., Nakano, H., Koga, M., and Ohshima, Y. (2003). The C. elegans che-1 gene encodes a zinc finger transcription factor required for specification of the ASE chemosensory neurons. Development *130*, 1215–1224. https://doi.org/10.1242/dev.00341.

58. Patel, T., and Hobert, O. (2017). Coordinated control of terminal differentiation and restriction of cellular plasticity. eLife *6*, e24100. https://doi.org/10.7554/eLife.24100.

59. Lans, H., Rademakers, S., and Jansen, G. (2004). A network of stimulatory and inhibitory Galpha-subunits regulates olfaction in Caenorhabditis elegans. Genetics *167*, 1677–1687. https://doi.org/10.1534/genetics.103.024786.

60. Perkins, L.A., Hedgecock, E.M., Thomson, J.N., and Culotti, J.G. (1986). Mutant sensory cilia in the nematode Caenorhabditis elegans. Dev. Biol. *117*, 456–487. https://doi.org/10.1016/0012-1606(86)90314-3.

61. Chalfie, M., and Sulston, J. (1981). Developmental genetics of the mechanosensory neurons of *Caenorhabditis elegans*. Dev. Biol. *82*, 358–370. https://doi.org/10.1016/0012-1606(81)90459-0.

62. Chang, A.J., Chronis, N., Karow, D.S., Marletta, M.A., and Bargmann, C.I. (2006). A Distributed Chemosensory Circuit for Oxygen Preference in C. elegans. PLOS Biol. *4*, e274. https://doi.org/10.1371/journal.pbio.0040274.

63. Gray, J.M., Karow, D.S., Lu, H., Chang, A.J., Chang, J.S., Ellis, R.E., Marletta, M.A., and Bargmann, C.I. (2004). Oxygen sensation and social feeding mediated by a C. elegans guanylate cyclase homologue. Nature *430*, 317–322. https://doi.org/10.1038/nature02714.

64. Zimmer, M., Gray, J.M., Pokala, N., Chang, A.J., Karow, D.S., Marletta, M.A., Hudson, M.L., Morton, D.B., Chronis, N., and Bargmann, C.I. (2009). Neurons detect increases and decreases in oxygen levels using distinct guanylate cyclases. Neuron *61*, 865–879. https://doi.org/10.1016/j.neuron.2009.02.013.

65. Onukwufor, J.O., Farooqi, M.A., Vodičková, A., Koren, S.A., Baldzizhar, A., Berry, B.J., Beutner, G., Porter, G.A., Belousov, V., Grossfield, A., et al. (2022). A reversible mitochondrial complex I thiol switch mediates hypoxic avoidance behavior in C. elegans. Nat. Commun. *13*, 2403. https://doi.org/10.1038/s41467-022-30169-y.

66. Berry, B.J., Baldzizhar, A., Nieves, T.O., and Wojtovich, A.P. (2020). Neuronal AMPK coordinates mitochondrial energy sensing and hypoxia resistance in C. elegans. FASEB J. *34*, 16333–16347. https://doi.org/10.1096/fj.202001150RR.

67. Bulteau, R., and Francesconi, M. (2022). Real age prediction from the transcriptome with RAPToR. Nat. Methods *19*, 969–975. https://doi.org/10.1038/s41592-022-01540-0.

68. Yu, S., Avery, L., Baude, E., and Garbers, D.L. (1997). Guanylyl cyclase expression in specific sensory neurons: A new family of chemosensory receptors. Proc. Natl. Acad. Sci. USA *94*, 3384–3387. https://doi.org/10.1073/pnas.94.7.3384.

69. Kaplan, J.M., and Horvitz, H.R. (1993). A dual mechanosensory and chemosensory neuron in Caenorhabditis elegans. Proc. Natl. Acad. Sci. USA *90*, 2227–2231. https://doi.org/10.1073/pnas.90.6.2227.

70. Colbert, H.A., Smith, T.L., and Bargmann, C.I. (1997). OSM-9, A Novel Protein with Structural Similarity to Channels, Is Required for Olfaction, Mechanosensation, and Olfactory Adaptation inCaenorhabditis elegans. J. Neurosci. *17*, 8259–8269. https://doi.org/10.1523/JNEUROSCI.17-21-08259.1997.

71. Tobin, D.M., Madsen, D.M., Kahn-Kirby, A., Peckol, E.L., Moulder, G., Barstead, R., Maricq, A.V., and Bargmann, C.I. (2002). Combinatorial Expression of TRPV Channel Proteins Defines Their Sensory Functions and Subcellular Localization in C. elegans Neurons. Neuron *35*, 307–318. https://doi.org/10.1016/S0896-6273(02)00757-2.

72. Rogers, C., Persson, A., Cheung, B., and de Bono, M. (2006). Behavioral Motifs and Neural Pathways Coordinating O2 Responses and Aggregation in C. elegans. Curr. Biol. *16*, 649–659. https://doi.org/10.1016/j.cub.2006.03.023.

73. Lord, T. (2025). Pathophysiological effects of hypoxia on testis function and spermatogenesis. Nat. Rev. Urol. *22*, 470–488. https://doi.org/10.1038/s41585-024-00969-6.

74. Toker, I.A., Ripoll-Sánchez, L., Geiger, L.T., Sussfeld, A., Saini, K.S., Beets, I., Vértes, P.E., Schafer, W.R., Ben-David, E., and Hobert, O. (2025). Divergence in neuronal signaling pathways despite conserved neuronal identity among *Caenorhabditis* species. Curr. Biol. *35*, 2927–2945.e7. https://doi.org/10.1016/j.cub.2025.05.036.

75. Ma, F., Lau, C.Y., and Zheng, C. (2021). Large genetic diversity and strong positive selection in F-box and GPCR genes among the wild isolates of Caenorhabditis elegans. Genome Biol. Evol. *13*, evab048. https://doi.org/10.1093/gbe/evab048.

76. de Bono, M., and Bargmann, C.I. (1998). Natural variation in a neuropeptide Y receptor homolog modifies social behavior and food response in C. elegans. Cell *94*, 679–689. https://doi.org/10.1016/s0092-8674(00)81609-8.

77. McGrath, P.T., Rockman, M.V., Zimmer, M., Jang, H., Macosko, E.Z., Kruglyak, L., and Bargmann, C.I. (2009). Quantitative mapping of a digenic behavioral trait implicates globin variation in C. elegans sensory behaviors. Neuron *61*, 692–699. https://doi.org/10.1016/j.neuron.2009.02.012.

78. Sterken, M.G., Snoek, L.B., Kammenga, J.E., and Andersen, E.C. (2015). The laboratory domestication of Caenorhabditis elegans. Trends Genet. *31*, 224–231. https://doi.org/10.1016/j.tig.2015.02.009.

79. Cornell, R., Cao, W., Liu, J., and Pocock, R. (2022). Conditional Degradation of UNC-31/CAPS Enables Spatiotemporal Analysis of Neuropeptide Function. J. Neurosci. *42*, 8599–8607. https://doi.org/10.1523/JNEUROSCI.1368-22.2022.

80. Zhao, Y., Long, L., Xu, W., Campbell, R.F., Large, E.E., Greene, J.S., and McGrath, P.T. (2018). Changes to social feeding behaviors are not sufficient for fitness gains of the Caenorhabditis elegans N2 reference strain. eLife *7*, e38675. https://doi.org/10.7554/eLife.38675.

81. Andersen, E.C., Bloom, J.S., Gerke, J.P., and Kruglyak, L. (2014). A Variant in the Neuropeptide Receptor npr-1 is a Major Determinant of Caenorhabditis elegans Growth and Physiology. PLOS Genet. *10*, e1004156. https://doi.org/10.1371/journal.pgen.1004156.

82. Petrella, L.N. (2014). Natural Variants of C. elegans Demonstrate Defects in Both Sperm Function and Oogenesis at Elevated Temperatures. PLOS One *9*, e112377. https://doi.org/10.1371/journal.pone.0112377.

83. Frézal, L., Saglio, M., Zhang, G., Noble, L., Richaud, A., and Félix, M.A. (2023). Genome-wide association and environmental suppression of the mortal germline phenotype of wild C. elegans. EMBO Rep. *24*, e58116. https://doi.org/10.15252/embr.202358116.

84. Chou, H.T., Valencia, F., Alexander, J.C., Bell, A.D., Deb, D., Pollard, D.A., and Paaby, A.B. (2024). Diversification of small RNA pathways underlies germline RNA interference incompetence in wild Caenorhabditis elegans strains. Genetics *226*, iyad191. https://doi.org/10.1093/genetics/iyad191.

85. Duveau, F., and Félix, M.-A. (2012). Role of Pleiotropy in the Evolution of a Cryptic Developmental Variation in Caenorhabditis elegans. PLOS Biol. *10*, e1001230. https://doi.org/10.1371/journal.pbio.1001230.

86. Gimond, C., Vielle, A., Silva-Soares, N., Zdraljevic, S., McGrath, P.T., Andersen, E.C., and Braendle, C. (2019). Natural Variation and Genetic Determinants of Caenorhabditis elegans Sperm Size. Genetics *213*, 615–632. https://doi.org/10.1534/genetics.119.302462.

87. Pollard, D.A., and Rockman, M.V. (2013). Resistance to Germline RNA Interference in a Caenorhabditis elegans Wild Isolate Exhibits Complexity and Nonadditivity. G3 (Bethesda) *3*, 941–947. https://doi.org/10.1534/g3.113.005785.

88. Ogawa, T., Yu, X., Shinohara, A., and Egelman, E.H. (1993). Similarity of the yeast RAD51 filament to the bacterial RecA filament. Science *259*, 1896–1899. https://doi.org/10.1126/science.8456314.

89. Alpi, A., Pasierbek, P., Gartner, A., and Loidl, J. (2003). Genetic and cytological characterization of the recombination protein RAD-51 in Caenorhabditis elegans. Chromosoma *112*, 6–16. https://doi.org/10.1007/s00412-003-0237-5.

90. Toraason, E., Adler, V.L., and Libuda, D.E. (2022). Aging and sperm signals alter DNA break formation and repair in the C. elegans germline. PLOS Genet. *18*, e1010282. https://doi.org/10.1371/journal.pgen.1010282.

91. Wu, Y., Wang, Y., Liu, Q., Zhu, L.J., Gao, H., Cui, M., Liu, J., Zhao, P., Liu, J., Chen, L., et al. (2019). Conserved microRNA mediates heating tolerance in germ cells versus surrounding somatic cells. RNA Biol. *16*, 1494–1503. https://doi.org/10.1080/15476286.2019.1639311.

92. Ito, H., Gaubert, H., Bucher, E., Mirouze, M., Vaillant, I., and Paszkowski, J. (2011). An siRNA pathway prevents transgenerational retrotransposition in plants subjected to stress. Nature *472*, 115–119. https://doi.org/10.1038/nature09861.

93. Subramanian, S. (2019). Little RNAs Go a Long Way: Long-Distance Signaling by MicroRNAs. Mol. Plant *12*, 18–20. https://doi.org/10.1016/j.molp.2018.12.001.

94. Jose, A.M. (2015). Movement of regulatory RNA between animal cells. Genesis *53*, 395–416. https://doi.org/10.1002/dvg.22871.

95. Félix, M.-A., and Wagner, A. (2008). Robustness and evolution: concepts, insights and challenges from a developmental model system. Heredity *100*, 132–140. https://doi.org/10.1038/sj.hdy.6800915.

96. Mestek Boukhibar, L., and Barkoulas, M. (2016). The developmental genetics of biological robustness. Ann. Bot. *117*, 699–707. https://doi.org/10.1093/aob/mcv128.

97. Tijsterman, M., May, R.C., Simmer, F., Okihara, K.L., and Plasterk, R.H.A. (2004). Genes Required for Systemic RNA Interference in Caenorhabditis elegans. Curr. Biol. *14*, 111–116. https://doi.org/10.1016/j.cub.2003.12.029.

98. Smendziuk, C.M., Messenberg, A., Vogl, W., and Tanentzapf, G. (2015). Bi-directional gap junction-mediated soma-germline communication is essential for spermatogenesis. Development *142*, 2598–2609. https://doi.org/10.1242/dev.123448.

99. Starich, T.A., Hall, D.H., and Greenstein, D. (2014). Two Classes of Gap Junction Channels Mediate Soma-Germline Interactions Essential for Germline Proliferation and Gametogenesis in Caenorhabditis elegans. Genetics *198*, 1127–1153. https://doi.org/10.1534/genetics.114.168815.

100. Marré, J., Traver, E.C., and Jose, A.M. (2016). Extracellular RNA is transported from one generation to the next in Caenorhabditis elegans. Proc. Natl. Acad. Sci. USA *113*, 12496–12501. https://doi.org/10.1073/pnas.1608959113.

101. Perez, M.F., and Lehner, B. (2019). Vitellogenins - Yolk Gene Function and Regulation in Caenorhabditis elegans. Front. Physiol. *10*, 1067. https://doi.org/10.3389/fphys.2019.01067.

102. Yemini, E., Lin, A., Nejatbakhsh, A., Varol, E., Sun, R., Mena, G.E., Samuel, A.D.T., Paninski, L., Venkatachalam, V., and Hobert, O. (2021). NeuroPAL: A Multicolor Atlas for Whole-Brain Neuronal Identification in *C. elegans*. Cell *184*, 272–288.e11. https://doi.org/10.1016/j.cell.2020.12.012.

103. O'Dea, R.E., Noble, D.W.A., Johnson, S.L., Hesselson, D., and Nakagawa, S. (2016). The role of non-genetic inheritance in evolutionary rescue: epigenetic buffering, heritable bet hedging and epigenetic traps. Environ. Epigenet. *2*, dvv014. https://doi.org/10.1093/eep/dvv014.

104. Sarkies, P. (2023). Evolution beyond DNA: epigenetic drivers for evolutionary change? BMC Biol. *21*, 272. https://doi.org/10.1186/s12915-023-01770-4.

105. Lenuzzi, M., Witte, H., Riebesell, M., Rödelsperger, C., Hong, R.L., and Sommer, R.J. (2023). Influence of environmental temperature on mouth-form plasticity in Pristionchus pacificus acts through daf-11-dependent cGMP signaling. J. Exp. Zool. Pt. B *340*, 214–224. https://doi.org/10.1002/jez.b.23094.

106. Sommer, R.J. (2020). Phenotypic Plasticity: From Theory and Genetics to Current and Future Challenges. Genetics *215*, 1–13. https://doi.org/10.1534/genetics.120.303163.

107. Teichman, G., Cohen, D., Ganon, O., Dunsky, N., Shani, S., Gingold, H., and Rechavi, O. (2023). RNAlysis: analyze your RNA sequencing data without writing a single line of code. BMC Biol. *21*, 74. https://doi.org/10.1186/s12915-023-01574-6.

108. Schindelin, J., Arganda-Carreras, I., Frise, E., Kaynig, V., Longair, M., Pietzsch, T., Preibisch, S., Rueden, C., Saalfeld, S., Schmid, B., et al. (2012). Fiji: an open-source platform for biological-image analysis. Nat. Methods *9*, 676–682. https://doi.org/10.1038/nmeth.2019.

109. Rual, J.-F., Ceron, J., Koreth, J., Hao, T., Nicot, A.-S., Hirozane-Kishikawa, T., Vandenhaute, J., Orkin, S.H., Hill, D.E., van den Heuvel, S., et al. (2004). Toward Improving Caenorhabditis elegans Phenome Mapping With an ORFeome-Based RNAi Library. Genome Res. *14*, 2162–2168. https://doi.org/10.1101/gr.2505604.

CellPress
OPEN ACCESS

110. Shahid, S., and Axtell, M.J. (2014). Identification and annotation of small RNA genes using ShortStack. Methods *67*, 20–27. https://doi.org/10.1016/j.ymeth.2013.10.004.

111. Liao, Y., Smyth, G.K., and Shi, W. (2014). featureCounts: an efficient general purpose program for assigning sequence reads to genomic features. Bioinformatics *30*, 923–930. https://doi.org/10.1093/bioinformatics/btt656.

112. Bray, N.L., Pimentel, H., Melsted, P., and Pachter, L. (2016). Near-optimal probabilistic RNA-seq quantification. Nat. Biotechnol. *34*, 525–527. https://doi.org/10.1038/nbt.3519.

113. Love, M.I., Huber, W., and Anders, S. (2014). Moderated estimation of fold change and dispersion for RNA-seq data with DESeq2. Genome Biol. *15*, 550. https://doi.org/10.1186/s13059-014-0550-8.

114. Ghanta, K.S., and Mello, C.C. (2020). Melting dsDNA Donor Molecules Greatly Improves Precision Genome Editing in Caenorhabditis elegans. Genetics *216*, 643–650. https://doi.org/10.1534/genetics.120.303564.

115. Wang, H., Park, H., Liu, J., and Sternberg, P.W. (2018). An Efficient Genome Editing Strategy To Generate Putative Null Mutants in Caenorhabditis elegans Using CRISPR/Cas9. G3 (Bethesda) *8*, 3607–3616. https://doi.org/10.1534/g3.118.200662.

STAR★METHODS

KEY RESOURCES TABLE

REAGENT or RESOURCE	SOURCE	IDENTIFIER
Antibodies		
Mouse anti-MSP	DSHB	RRID: AB_760348
Rabbit anti-RAD-51	Gift from Nicolas Silva	N/A
Cy™3 AffiniPure® Donkey Anti-Mouse IgG	Jackson ImmunoResearch	RRID: AB_2315777
Cy™3 AffiniPure® Donkey Anti-Rabbit IgG (H+L)	Jackson ImmunoResearch	RRID: AB_2307443
Bacterial and virus strains		
OP50	Caenorhabditis Genetics Center	N/A
HT115	Caenorhabditis Genetics Center	N/A
Chemicals, peptides, and recombinant proteins		
Alt-R™ S.p. Cas9 Nuclease V3	IDT	Cat#1081058
Critical commercial assays		
NEBNext® Small RNA Library Prep Set for Illumina®	New England Biolabs	Cat#E7330L
NEBNext® Ultra™ II Directional RNA Library Prep Kit for Illumina®	New England Biolabs	Cat#E7760, #E7765
Deposited data		
Raw and analyzed sequencing data	This paper	GEO: GSE331410
AGO IP sequencing data	Seroussi et al.[37]	GEO: GSE208702
rde-4 RNA sequencing data	Posner et al.[43]	GEO: GSE124049
Experimental models: Organisms/strains		
C. elegans strain: WM48 rde-4(ne299) III	Caenorhabditis Genetics Center	WM48
C. elegans strain: BFF337 rde-4(ne299) III (3X BC to N2)	This study	BFF337
C. elegans strain: N2	Caenorhabditis Genetics Center	WB Strain: N2
C. elegans strain: BFF496 rde-4(pig51) III	This study	BFF496
C. elegans strain: BFF497 rde-4(pig52) III	This study	BFF497
C. elegans strain: BFF499 LSJ1; rde-4(pig53) III	This study	BFF499
C. elegans strain: BFF509 CB4856; rde-4(pig54) III	This study	BFF509
C. elegans strain: BFF504 rde-4(ne299) III; kyIR1 (V,CB4856>N2) V; qqIR1(X,CB4856>N2) X	This study	BFF504
C. elegans strain: BFF505 rde-4(ne299) III; qqIR1(X,CB4856>N2) X	This study	BFF505
C. elegans strain: DR466 him-5(e1490) V	Caenorhabditis Genetics Center	WB Strain: DR466
C. elegans strain: BFF401 rde-4(ne299) III; him-5(e1490) V	This study	BFF401
C. elegans strain: JK574 fog-2(q71) V	Caenorhabditis Genetics Center	WB Strain: JK547
C. elegans strain: BFF16 pigSi3[Psng-1::rde-4:SL2::yfp+cb-unc-119(+)] II; rde-4(ne299)	Posner et al.[43]	BFF16
C. elegans strain: BFF397 rde-4(ne299) III; sid-1(qt78) V	This study	BFF397
C. elegans strain: BFF431 pigSi3[Psng-1::rde-4::SL2:yfp+cb-unc-119(+)] II; rde-4(ne299) III; sid-1(qt78) V	This study	BFF431

(*Continued on next page*)

Continued

REAGENT or RESOURCE	SOURCE	IDENTIFIER
C. elegans strain: BFF430 tph-1(mg280) II; rde-4(ne299) III	This study	BFF430
C. elegans strain: BFF438 mec-8(ok2043) I; rde-4(ne299) III	This study	BFF438
C. elegans strain: BFF468 rde-4(ne299) III; osm-9(ky10) IV	This study	BFF468
C. elegans strain: BFF476 rde-4(ne299) III; gpa-3(pk35) odr-3(n1605) V.	This study	BFF476
C. elegans strain: BFF477 che-1(luc174) I; rde-4(ne299) III	This study	BFF477
C. elegans strain: BFF487 rde-4(ne299) III; npr-1(ad609)X	This study	BFF487
C. elegans strain: BFF485 pigSi3[Psng-1::rde-4:SL2::yfp+cb-unc-119(+)] II; rde-4(ne299)III; qIs147 [sur-5::GFP] IV	This study	BFF485
C. elegans strain: BFF486 rde-4(ne299) III; qIs147 [sur-5::GFP] IV	This study	BFF486
C. elegans strain: BFF489 gcy-35(ok769) I; rde-4(ne299) III	This study	BFF489
C. elegans strain: BFF491 rde-4(ne299) III; gcy-33(ok232) V	This study	BFF491
C. elegans strain: BFF531 rde-4(ne299) III; peIs1713 [sra-6p::mCasp-1 + unc-122p::mCherry]	This study	BFF531
C. elegans strain: BFF362 rde-4(ne299) III; cmk-1(oy21) IV	This study	BFF362
C. elegans strain: BFF419 tax-2(ok3403) I; rde-4(ne299) III	This study	BFF419
C. elegans strain: BFF423 rde-4(uu53) III; him-5(e1490) V	This study	BFF423
C. elegans strain: BFF463 rde-4(ne299) III; egl-21(n476) IV; him-5(e1490) V	This study	BFF463
C. elegans strain: JK4846 qIs147 [sur-5::GFP] IV	Caenorhabditis Genetics Center	JK4846
C. elegans strain: PD2227 oxIs322 [myo-2p::mCherry::H2B + myo-3p::mCherry::H2B + Cbr-unc-119(+)] II. ccTi1594 [mex-5p::GFP::gpr-1::smu-1 3′UTR + Cbr-unc-119(+), III: 680195] III	Caenorhabditis Genetics Center	WB Strain: PD2227
C. elegans strain: BFF429 mjIs134 [mex-5p::gfp::h2b::tbb-2] II; rde-4(ne299) III	This study	BFF429
C. elegans strain: SX1263 mjIs134 [mex-5p::gfp::h2b::tbb-2] II	Caenorhabditis Genetics Center	SX1263
C. elegans strain: BFF467 rde-4(ne299) III; alg-3(tm1155) IV; him-5(e1490)V	This study	BFF467
C. elegans strain: AMJ912 jamSi28[Pmyo-3::rde-4(+)::rde-4 3′UTR] II; rde-4(ne301) III	Caenorhabditis Genetics Center	WB Strain: AMJ912
C. elegans strain: AMJ565 jamSi6 [Pnas-9::rde-4(+)::rde-4 3′UTR] II; rde-4(ne301) III; unc-119(ed3) III (?)	Caenorhabditis Genetics Center	AMJ565
C. elegans strain: WM49 rde-4(ne301)	Caenorhabditis Genetics Center	WB Strain: WM49
C. elegans strain: BFF555 reSi7 I [rgef-1p::TIR1::F2A::mTagBFP2::AID*::NLS::tbb-2 3′UTR] (I: 5.32); rde-4(ne299) III; unc-31(rp166[GFP::TEV::AID*::FLAG::unc-31]) IV	This study	BFF555
C. elegans strain: BFF554 tax-2(p694) I; rde-4(ne299) III	This study	BFF554
C. elegans strain: BFF572 gcy-35(db2020(gcy-35[framshift+stop codon])) I; rde-4 (ne299) III (line 1) (2X BC)	This study	BFF572
C. elegans strain: BFF573 gcy-35(db2020(gcy-35[framshift+stop codon])) I; rde-4 (ne299) III (line 2) (2X BC)	This study	BFF573
C. elegans strain: BFF563 gcy-35(db2020(gcy-35[framshift+stop codon])) I; npr-1(ok1447) X	This study	BFF563
C. elegans strain: RB1330 npr-1(ok1447) X	Caenorhabditis Genetics Center	WB Strain: RB1330
C. elegans strain: BFF523 npr-1(ok1447) X (2X BC of RB1330)	This study	BFF523
C. elegans strain: BFF542 rde-4(ne299) III; npr-1(ok1447) X	This study	BFF542
C. elegans strain: BFF547 rde-4(pig51) III; flp-19(ok2460) X	This study	BFF547
C. elegans strain: BFF550 rde-4(pig51) III; flp-21(ok889) V	This study	BFF550
C. elegans strain: BFF556 rde-4(pig51) III; flp-8(pk360) X	This study	BFF556

(*Continued on next page*)

Continued		
REAGENT or RESOURCE	SOURCE	IDENTIFIER
C. elegans strain: PHX11501 rde-4(syb11410 syb11501)	This study/SunyBiotech	PHX11501
C. elegans strain: BFF574 bmdSi348 [loxN::rgef-1p::FLP::P2A::H2B::2xmTurq2] I; rde-4(syb11410 syb11501) III	This study	BFF572
Oligonucleotides		
rde-4 Alt-R gRNA #1:ACTTGGGGCACTGTCGAACT	IDT	N/A
rde-4 Alt-R gRNA #2: ATCTCTGGAATCATATGATA	IDT	N/A
rde-4 Alt-R HDR donor block: AGGCTGCTAAGGCT GTCTATCAAAAGACGCCAACTATATGGGTATGCCT CCAAATAATTGTAGTTAATAT	IDT	N/A
gcy-35 Alt-R gRNA: GCATGATTCTCACAACGCTC	IDT	N/A
gcy-35 Alt-R HDR donor block: GTGATGCCTATATGATTGTGG GCGGAGTTCCGGAGGGGAAG TTTGTCCAGAGCAGAGGTGAC TAAGTGATAAGCTAGCCGTTGT GAGAATCATGCAGAGCGAGTTCTCAATAT	IDT	N/A
Recombinant DNA		
Plasmid: pL4440-RNAi control (HT115)	Vidal RNAi library	N/A
Plasmid: pL4440-gfp (HT115)	Vidal RNAi library	N/A
Software and algorithms		
RNAlysis	Teichman et al.[107]	https://github.com/GuyTeichman/RNAlysis
ImageJ	Schindelin et al.[108]	https://imagej.net/ij/
GraphPad Prism 10	GraphPad	https://www.graphpad.com/
R/RStudio	R Core Team	https://www.r-project.org/
BioRender	BioRender	https://www.biorender.com/
DoubleBlind	Teichman et al.[56]	https://github.com/GuyTeichman/DoubleBlind
Inkscape v1.4	Inkscape	https://inkscape.org/
Zentracker	de Bono lab	https://github.com/wormtracker/zentracker; RRID SCR_022006

EXPERIMENTAL MODEL AND STUDY PARTICIPANT DETAILS

C. elegans strains were maintained on standard Nematode Growth Medium (NGM) plates seeded with *E. coli* OP50 at 20°C, unless stated otherwise. A complete list of strains used in this study is provided in the key resources table. Note that *ne301* and *ne299* alleles contain an identical lesion (see WBVar00090971)[27,28]; we used the original designations to preserve a clear record.

METHOD DETAILS

Fertility assay

L4 animals, identified by the characteristic white crescent surrounding the developing vulva, were transferred to 25°C. After ~24 hours, individual day 1 adults were moved to fresh NGM plates seeded with a small (~30µL) drop of OP50 that had been allowed to dry for 1–2 days. About 12 hours later, the number of laid eggs and unfertilized oocytes was quantified and the percent of unfertilized oocytes was calculated. Samples with fewer than 20 total eggs and unfertilized oocytes were excluded from analysis. For *egl-21(n476)* mutants, which display egg-laying defects, counts from three biological replicates were pooled. In all cases, the investigators were blinded to the genotypes.

Fertility assay on uniform bacterial lawn

To create uniform lawns, 40µL of OP50 bacteria was added onto 30mm NGM plates and spread evenly across the entire plate surface with a bacterial spreader. On the same day, an equal volume was seeded as a small drop on separate plates to serve as the standard/small lawn control. Plates were allowed to dry for 1–2 days and fertility assays were performed as described above.

RNAi

RNAi feeding clones were obtained from the Vidal library.[109] The bacteria were inoculated overnight at 37°C in Lysogeny broth (LB) containing 50μg/ml carbenicillin. 1mM of IPTG was then added to the bacterial culture and 50–100μL was seeded onto 60mm NGM agar plates containing 1mM IPTG and 25μg/mL carbenicillin. After the seeded plates were dried for ~24 hours, 5–10 L4 animals were placed on the RNAi plates. The progeny was then collected for analyses.

AID-Mediated degradation of UNC-31

NGM plates were supplemented with 1mM auxin (indole-3-acetic acid) prepared from a stock of 400mM dissolved in ethanol (EtOH). NGM plates supplemented with EtOH were used as a control. Auxin solution and auxin-containing plates were protected from light at all time. Mid-L4 animals (BFF555: *reSi7 I [rgef-1p::TIR1::F2A::mTagBFP2::AID*::NLS::tbb-2 3′UTR] (I: 5.32); rde-4(ne299)* III*; unc-31(rp166[GFP::TEV::AID*::FLAG::unc-31]) IV*) were transfer to seeded NGM plates containing auxin or EtOH and shifted to 25°C. Fertility was assessed as described above.

RNA isolation

Total RNA was isolated from day-1 adults (~72 hours at 20°C following L1 synchronization) using standard phenol-chloroform method with TRIzol™ (Invitrogen). RNA quality was accessed using Agilent 4150 BioAnalyzer instrument and High Sensitivity RNA ScreenTapes.

RNA-seq

To sequence the mRNA, RNA libraries were prepared using NEBNext® Ultra II Directional RNA Library Prep Kit for Illumina® coupled with NEBNext® poly(A) mRNA Magnetic Isolation Module. The cDNA libraries were pooled and paired-end sequencing was performed on the NextSeq 2000 platform.

For sRNA sequencing, we treated the RNA samples with RNA 5′ polyphosphatase (epicentre) and the libraries were prepared using NEBNext® Multiplex Small RNA Library Prep Set for Illumina (New England Biolabs) or TruSeq Small RNA Library Prep Kit (Illumina) according to the manufacturer's protocol. RNA ranging from ~140 to 160nt was size-selected by gel extraction using 4% agarose E-Gel (Life Technologies). The pooled samples were the sequenced using the Illumina HiSeq 2500 instrument.

Bioinformatics

All bioinformatic analyses were performed using RNAlysis.[107] For sRNA-seq data, sRNA reads were aligned to PRJNA13758 CE11 genome assembly using ShortStack[110] and aligned anti-sense reads were quantified using FeatureCounts (Data S2).[111] We included all reads with > 5 RPM without imposing any constraint on the length and 5′ nucleotide. For mRNA, we pseudo-aligned reads using Kallisto (Data S3).[112] We then performed differential expression analysis using DESeq2.[113] For gene set enrichment analysis, $\log_2$ (fold enrichment) scores were computed and the FDR for enrichment was calculated using 10,000 random gene sets identical in size to the tested group. For Gene Ontology (GO) and KEGG pathways analyses, Fisher's exact tests were performed.

CRISPR/Cas9-mediated knockout of *rde-4*

CRISPR/Cas9 was performed as previously described.[114] To generate *rde-4* deletion, we used two crRNAs: ACTTG GGGCACTGTCGAACT and ATCTCTGGAATCATATGATA. The following homology-directed repair (HDR) donor was used: AGGCTGCTAAGGCTGTCTATCAAAAGACGCCAACTATATGGGTATGCCTCCAAATAATTGTAGTTAATAT. This generates 637bp deletion in *rde-4*. The results strains were backcrossed at least two times to remove potential background mutations.

CRISPR/Cas9-mediated knockout of *gcy-35*

A 43bp STOP-IN cassette, containing stop codons in all three reading frames and cause frameshift,[115] was inserted into *gcy-35* in *rde-4(ne299)* (BFF337). The sequences of the crRNA and HDR donor were GCATGATTCTCACAACGCTC and GTGATGCCT ATATGATTGTGGGCGGAGTTCCGGAGGGGAAGTTTGTCCAGAGCAGAGGTGACTAAGTGATAAGCTAGCCGTTGTGAGAATCATG CAGAGCGAGTTCTCAATAT, respectively. The results strains were backcrossed twice to remove potential background mutations.

Low oxygen exposure experiment

Wild-type and *rde-4(ne299)* animals carrying germline-specific transgene *mjIs134 [mex-5p::gfp::h2b::tbb-2]* were maintained under normoxia at 20°C before the experiments. L4 worms were picked onto several NGM plates seeded with OP50. Half the plates were placed in a glove box (Coy Laboratory Products) maintained at either 7% or 1% oxygen (balanced with nitrogen) at 25°C for 24 hours. Control plates were placed in a 25°C incubator under normoxia (~21% oxygen) for the same duration. Imaging was then performed on day-1 adults using Nikon Ti2E Imaging System with a Plan Apo λ 20x/0.75 DIC 1 air PFS objective. Their germline morphology – the accumulation of unfertilized oocytes ("stacked") accompanied by the absence of fertilized embryos in the proximal gonad – was scored blind using Fiji[108] and DoubleBlind (https://github.com/GuyTeichman/DoubleBlind). Note that this assay avoids the plate transfers used in our standard protocol (see above) and therefore minimizes fluctuations in oxygen levels.

Locomotion assays

60µl of OP50 was seeded on low peptone NGM plates (0.13% wt/vol bactopeptone) 2 days before the assay. L4 animals were picked 24 hours before the experiment and incubated at 25 or 20°C. A rubber stamp was used to remove the edge of the bacterial lawn on the day of the assay. 20–25 animals were picked onto the lawn and left for 10mins before starting the assay. A 10 x 10 x 0.4 mm PDMS chamber was placed on top of the bacterial lawn and defined gas mixtures were delivered to the chamber at 1.25ml/min using a syringe pump (PHD 2000, Harvard Apparatus). Before recording behavior, we acclimated animals to 7 % oxygen for 2 mins. For standard assays, animals were exposed to 2 mins of 7%, 2 mins of 21%, and 2 mins of 7% oxygen. Video recordings were acquired at 2 frames per second (fps) using a Grasshopper camera (Point Grey) mounted on a stereomicroscope (Leica MZ6 or MZ7.5). Videos were analyzed and animal speed was calculated using custom-written MATLAB software (Zentracker: https://github.com/wormtracker/zentracker; RRID SCR_022006). Average speed values were extracted using Metaverage, a custom-written MATLAB software. Average speed during 30 second intervals at the end of the first 2 mins at 7% and at the end of the 21% oxygen period were used for statistical comparisons.

Antibody and DAPI staining

All experiments were performed on day-1 adults. For experiments at 25°C, L4 animals were shifted to 25°C as described above. Dissected gonads were fixed with 3.5% formaldehyde for 10 mins and washed at least twice with PBS-T (0.1% Tween-20 in PBS buffer). Tissues were then permeabilized in 0.25% PBS-Triton for 20 mins, followed by blocking in 0.5% BSA in PBS-T for 1 hour with gentle shaking. Next, primary antibodies – mouse anti-MSP (Developmental Studies Hybridoma Bank) and rabbit anti-RAD-51 (gift from Nicolas Silva) – were diluted 1:1000 in PBS-T and applied for 1 hour with gentle agitation. After two washes with PBS-T, secondary antibodies (Jackson ImmunoResearch anti-rabbit and anti-mouse; 1:200 in PBS-T) were added and incubated for 1 hour. Samples were then washed for two more times and were incubated with DAPI (0.02% in PBS-T) for 10 mins, followed by two more washes with PBS-T. Finally, samples were mounted on glass slides in VECTASHIELD® Antifade Mounting Medium (Vector Laboratories, Cat# H-1000) prior to imaging.

QUANTIFICATION AND STATISTICAL ANALYSIS

Statistical analyses were conducted using R software v4.2.3 and GraphPad Prism 10. For comparisons between two groups, non-parametric unpaired Wilcoxon rank sum tests were used, unless stated otherwise. For experiments involving multiple groups, Kruskal-Wallis test followed by pairwise Wilcoxon rank-sum tests were performed. Multiple comparison corrections using the Benjamini-Hochberg method were applied where appropriate.

Current Biology, Volume *36*

Supplemental Information

Neuronal RNAi and oxygen-sensing circuit shape germline resilience to heat stress

Chee Kiang Ewe, Hanna Achache, Hanna Schön, Leonid Kontorovich, Guy Teichman, Shir Weiss, Anna Mogilevskaya, Myriam Valenski, Sarit Anava, Rutwik Bardapurkar, Hila Gingold, Rachel Posner, Olga Antonova, Mario de Bono, Yonatan B. Tzur, and Oded Rechavi

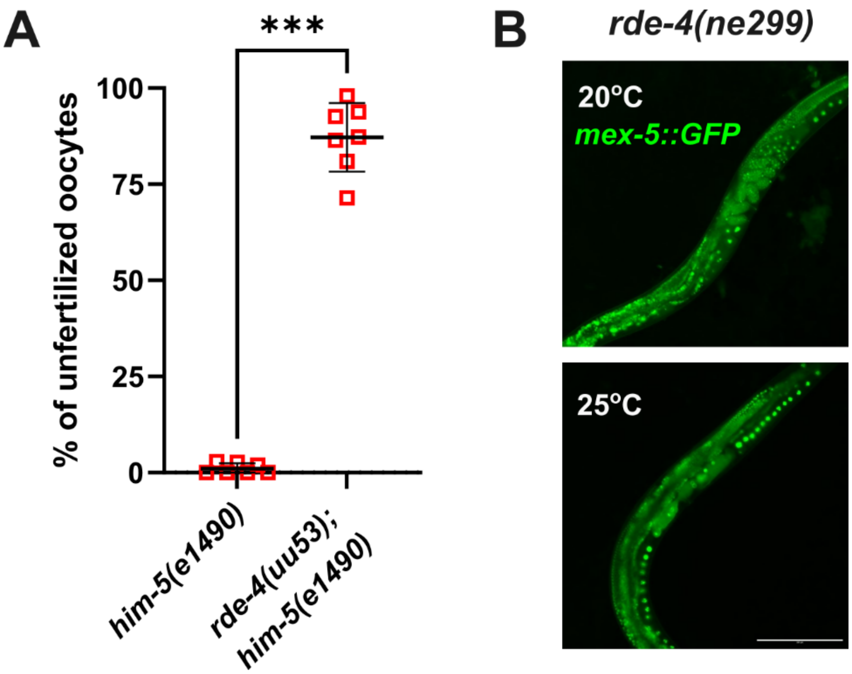

Figure S1. RDE-4 promotes reproduction at high temperature. Related to Figure 1. (**A**) *rde-4(uu53)* mutants exhibit severe loss of fertility at 25 °C. Error represents mean ± SD. Statistical significance was determined by Mann-Whitney test. *** $p < 0.001$. (**B**) *rde-4(ne299)* day 2 adult shows accumulation of unfertilized oocyte stacked in the gonad at 25 °C, but not at 20 °C. The germline is marked by *gfp* driven by *mex-5* (RNA-Pol II) promotor.

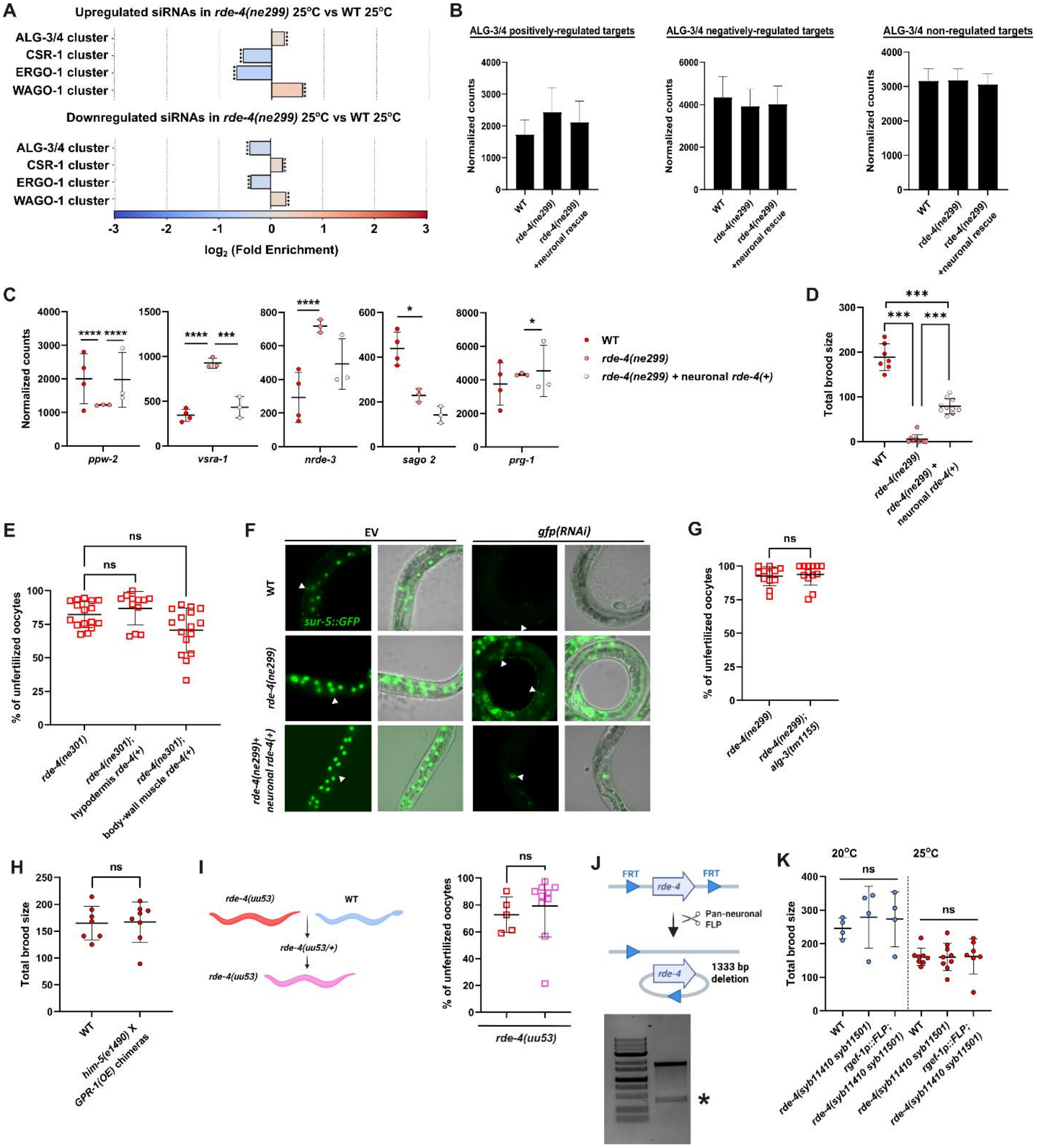

Figure S2. Neuronal RDE-4 promotes sperm development. Related to Figure 2. (**A**) Different classes of endo-siRNAs are differentially expressed in *rde-4(ne299)*. ALG-3/4-class siRNAs tend to be upregulated, whereas CSR-1–class siRNAs tend to be downregulated in *rde-4(ne299)* compared to wild type. (**B**) Expression of different classes of ALG-3/4 targets, as defined by Conine et al, in wild type, *rde-4(ne299)*, and *pigSi3(Psng-1::rde-4); rde-4(ne299)* mutants. Error represents mean ± SEM. (**C**) Misexpression of AGO genes in *rde-4(ne299)* is rescued by neuronal *rde-4(+)* in some cases. Relative log expression (RLE) is shown. Statistical significance was determined by DESeq2. (**D**) *rde-4(ne299)* mutants show a reduced brood size compare with

wild type, and this is rescued by *pigSi3(Psng-1::rde-4)*.(**E**) Expressing *rde-4* in hypodermis (driven by *nas-9* promotor) or body-wall muscle (drive by *myo-3* promotor) does not rescue *rde-4(ne301)* fertility defects. Note that *ne301* and *ne299* contain identical lesion. (**F**) Neuronal *rde-4(+)* does not rescue RNAi-defective phenotype of *rde-4(ne299)*. Arrows indicate spermatheca. (**G**) Loss of *alg-3* does not affect *rde-4(ne299)* fertility. These strains contain *him-5(e1490)* mutation. (**H**) F1 chimeras generated by crossing GPR-1-overexpressing hermaphrodites with *him-5(e1490)* males does not show altered brood size. (**I**) Homozygous *rde-4(uu53)* segregated from heterozygous mothers show severe fertility defects. (**J**) Schematic diagram depicting FRT/FLP-mediated excision of neuronal *rde-4*. Asterisk denotes the deleted *rde-4* allele identified by PCR. (**K**) Deleting *rde-4* in neurons using the FRT/FLP system does not affect brood size. For (D), (E), (G), (H), (I), and (K), error represents mean ± SD and statistical significance was determined by Mann-Whitney tests. Multiple comparison corrections were applied where appropriate. ns $p > 0.05$; * $p \leq 0.05$; *** $p < 0.001$; **** $p < 0.0001$.

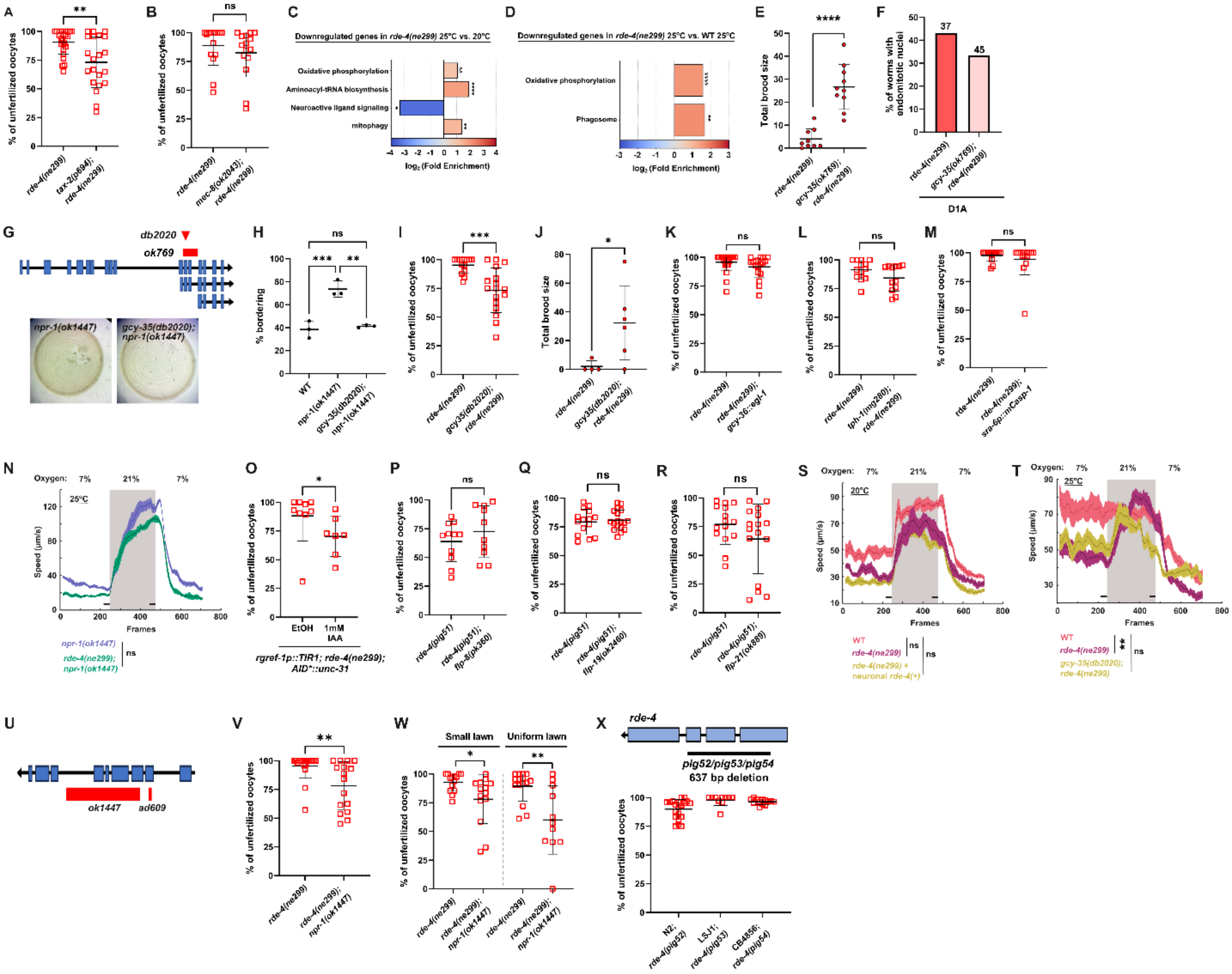

Figure S3. Neuronal sensory and NPR-1 affects fertility. Related to Figure 3. (**A**) Loss of *tax-2* partially rescues fertility defects in *rde-4(ne299)* at 25 °C. (**B**) Knocking out *mec-8* does not affect *rde-4(ne299)* fertility. (**C and D**) KEGG pathway analysis reveals that mitochondrial genes tend to be downregulated in *rde-4(ne299)* grown at 25 °C compared to either *rde-4(ne299)* at 20 °C or wild type at 25 °C. Differential gene expression analysis includes RAPToR age estimates as covariate. (**E and F**) Deleting *gcy-35* increases brood size and reduces the accumulation of endomitotic nuclei. D1A = day-1 adults. (**G and H**) Inserting a universal knock-in cassette (*db2020*) causes loss of function of *gcy-35*, which inhibits aerotaxis behavior of *npr-1(ok1447)*. (**I and J**) *gcy-35(db2020)* rescues fertility defects in *rde-4(ne299)*. (**K**) Ablating *gcy-36*-expressing neurons does not affect *rde-4(ne299)* fertility. (**L**) Eliminating *tph-1* does not rescue *rde-4(ne299)* sterility. (**M**) Genetically ablating ASH neurons does not impact *rde-4(ne299)* fertility. (**N**) *npr-1(ok1447)* mutants show increased responses to 7 % and 21 % oxygen at 25 °C. This is not affected by the loss of *rde-4*. (**O**) Depleting UNC-31 by AID partially restore *rde-4(ne299)* fertility. (**P-R**) Knocking out *flp-8*, *flp-19*, or *flp-21* does not affect *rde-4(pig51)* fertility. Error represents mean ± SD. (**S**) *rde-4(ne299)* and *pigSi3(Psng-1::rde-4); rde-4(ne299)* mutants show similar oxygen response as wild type at 20 °C. (**T**) Loss of *gcy-35* rescues oxygen response of *rde-4(ne299)*. (**U**) Schematic diagram depicting the positions and molecular nature of the *ok1447* and *ad609* mutations in *npr-1*. (**V**) Deleting *npr-1* partially rescues *rde-4(ne299)* fertility. (**W**) Deletion of *npr-1* rescues *rde-4(ne299)* fertility on both small

‘simple’ lawns and uniform lawns. (**X**) Deleting *rde-4* in CB4856 and LSJ1 causes severe loss of fertility at 25 ^{o}C, similar to that observed in N2. Error represents mean ± SD. For (N), (S), and (T), n = 7-9 assays, 20-25 animals per assay. Solid lines indicate average speed and error indicates SEM. Black horizontal bars indicate time intervals used for statistical tests. For (A), (B), (E), (H-T), (V) and (W), statistical significance was determined by Mann-Whitney tests. Multiple comparison corrections were applied where appropriate. ns $p > 0.05$; * $p \leq 0.05$; ** $p < 0.01$; p *** < 0.001; **** $p < 0.0001$.

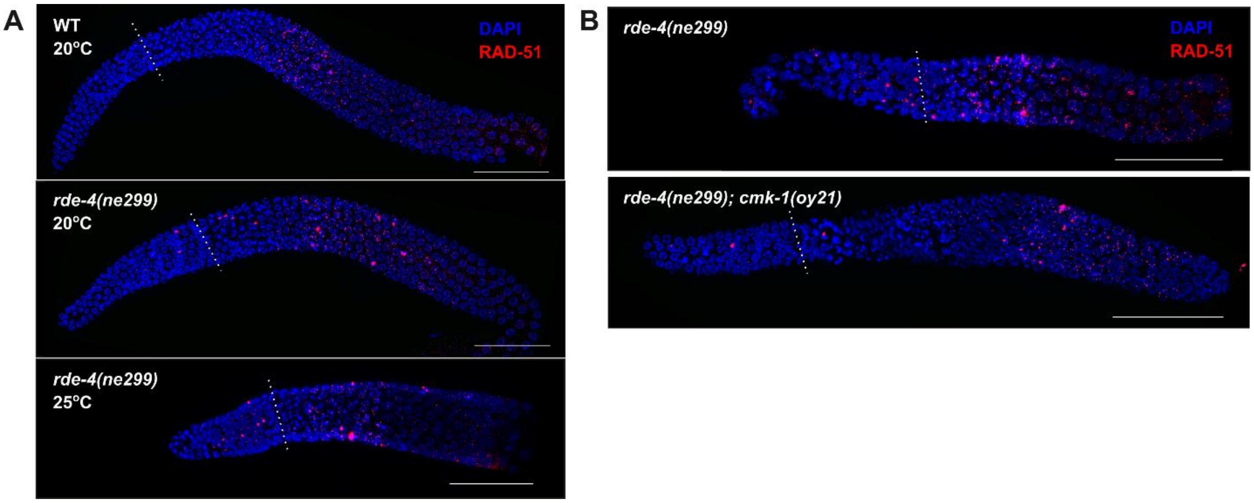

Figure S4. Neuronal sensory affects germline integrity. Related to Figure 4. (**A**) *rde-4(ne299)* grown at 25 ^{o}C, but not 20 ^{o}C, shows increased DSBs in the gonad. (**B**) Knocking out *cmk-1* reduces DSBs in *rde-4(ne299)* at 25 ^{o}C. White dotted lines indicate mitosis to meiosis transition. Antibody staining was performed on day-1 adults. Scale bar = 50 µm.
